# Functional characterization of a catalytically promiscuous tryptophan decarboxylase from camptothecin-producing *Camptotheca acuminata*

**DOI:** 10.3389/fpls.2022.987348

**Published:** 2022-08-18

**Authors:** Chong Qiao, Fei Chen, Zhan Liu, Tianfang Huang, Wei Li, Guolin Zhang, Yinggang Luo

**Affiliations:** ^1^Center for Natural Products Research, Chengdu Institute of Biology, Chinese Academy of Sciences, Chengdu, China; ^2^University of Chinese Academy of Sciences, Beijing, China

**Keywords:** tryptophan decarboxylase, promiscuity, tryptamine, decarboxylation, *Camptotheca acuminata*

## Abstract

Tryptophan decarboxylases (TDCs) are a group of pyridoxal 5′-phosphate-dependent enzymes involved in the enzymatic conversion of tryptophan into tryptamine, a critical biogenic amine. We herein mined and cloned a TDC-encoding gene, *CaTDC3*, from camptothecin-producing plant *Camptotheca acuminata*. The intact *CaTDC3* was heterologously overexpressed in *Escherichia coli* and the recombinant CaTDC3 was purified to homogeneity. High-performance liquid chromatography (HPLC)-diode array detector (DAD) and high resolution mass spectrometry (HRMS) data analyses of the CaTDC3-catalyzed reaction mixture confirmed the catalytically decarboxylative activity of CaTDC3. CaTDC3 shows strict stereoselectivity for L-tryptophan. Homology modeling and molecular docking implied CaTDC3’s recognition of L-tryptophan derivatives and analogs. Substrate scope investigations revealed that the appropriate substituent groups on the indole ring, i.e., hydroxylated and halogenated L-tryptophans, could be recognized by CaTDC3 and the decarboxylation reactions generated the corresponding tryptamines. The C^β^ -methyl-L-tryptophans were decarboxylated by CaTDC3 efficiently. 1-Thio-L-tryptophan, the NH group of the indole ring replaced by an S atom, could be decarboxylated by CaTDC3. CaTDC3 catalyzed the decarboxylation of 7-aza-L-tryptophan, an N displacement of the C on the aromatic ring, to afford 7-aza-tryptamine. L-Kynurenine, an L-tryptophan degradation product, could be decarboxylated by CaTDC3. The present works uncover a catalytically promiscuous TDC and the TDC is a versatile decarboxylase in synthetic biology for specialized pharmaceutically important substances.

## Introduction

**FIGURE 1:**
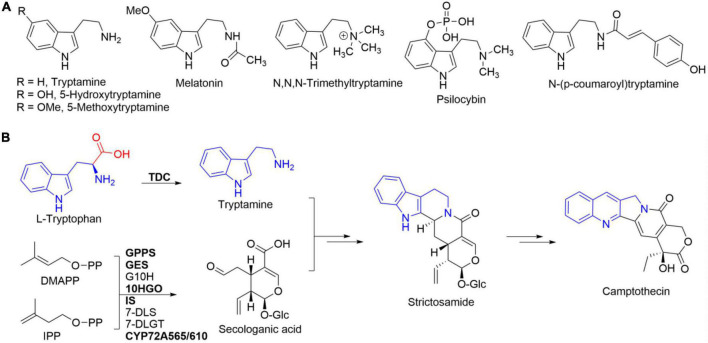
Tryptamine and its representative analogs **(A)** and L-tryptophan’s integration into camptothecin *via* tryptamine **(B)**. Tryptamine is originated from the decarboxylation of L-tryptophan catalyzed by tryptophan decarboxylase (TDC). Enzymes in bold represented those that have been functionally characterized.

Tryptamine (Figure 1A), an essential biogenic amine generated from the decarboxylation reaction of tryptophan, plays a fundamental role in both primary and secondary metabolisms in all living organisms (Wang et al., 2019; Negri et al., 2021). Tryptamine, produced by the microbes living in human gut, was reported to accelerate their whole-gut transit time (Negri et al., 2021). Tryptamine had been verified to act as an important compound for interspecific communication in some entomopathogenic fungi to kill their hosts (Bhattarai et al., 2018). Tryptamine and its derivatives (Figure 1A), including 5-hydroxytryptamine (serotonin), 5-methoxytryptamine, and melatonin, are well-known modulators/regulators in both animals and plants (Wang et al., 2019; Negri et al., 2021). Additionally, tryptamine is a key entry into the biosynthetic pathways for pharmaceutically important specialized metabolites such as camptothecin (Figure 1B), ajmalicine, and vincristine (O’Connor and Maresh, 2006; Negri et al., 2021).

Tryptamine has been frequently used as a chemical building block for the synthesis of many biologically and pharmaceutically active compounds (Righi et al., 2012). The methylated tryptamine derivative N,N,N-trimethyltryptamine (Figure 1A) had been reported to be related to the plant defense against herbivory (Servillo et al., 2013). Psilocybin (Figure 1A), 4-phosphorylated tryptamine, showed a positive trend in the treatment of anxiety with advanced-stage cancer patients and for nicotine addiction in clinical study (Fricke et al., 2017). In addition, the hydroxycinnamoylated tryptamine, e.g., N-(*p*-coumaroyl)tryptamine, exhibited antioxidant, anti-inflammatory, and anti-atherogenic activities (Lee et al., 2017).

Tryptophan decarboxylases (TDCs), a group of pyridoxal 5′-phosphate (PLP)-dependent enzymes, are in charge of the enzymatic conversion of tryptophan into tryptamine (Figure 1B; López-Meyer and Nessler, 1997; O’Connor and Maresh, 2006). The first plant TDC-encoding gene had been isolated from *Catharanthus roseus*, the antitumor monoterpene indole alkaloid vincristine-producing plant (De Luca et al., 1989). Based on homology cloning strategy, a few *TDC* genes had been cloned from different plants such as *Ophiorrhiza pumila*, *Oryza sativa*, *Capsicum annuum*, and *Rauvolfia verticillata* (Yamazaki et al., 2003; Kang et al., 2007; Park et al., 2009; Liu et al., 2012). One TDC-encoding gene was cloned and characterized from *R. verticillata* (Liu et al., 2012). Two TDC-encoding genes, *CanTDC1* and *CanTDC2*, were isolated from pepper fruits (*C. annuum*) (Park et al., 2009). The specific catalytic activity of recombinant CanTDC1 was three times higher than that of CanTDC2 (Park et al., 2009). Besides, the expression of *CanTDC1* was highly induced by elicitors, whereas *CanTDC2* was constitutively expressed at low level in all pepper tissues (Park et al., 2009). Seven *TDC* genes were reported in the genome of rice (*O. sativa*) (Kang et al., 2007). However, only two genes, *OsTDCAK31* and *OsTDCAK53*, were successfully expressed in *Escherichia coli* and their catalytic decarboxylation activities toward L-tryptophan were confirmed (Kang et al., 2007). These results revealed the presence of one or more TDC-encoding genes in one plant species.

**FIGURE 2:**
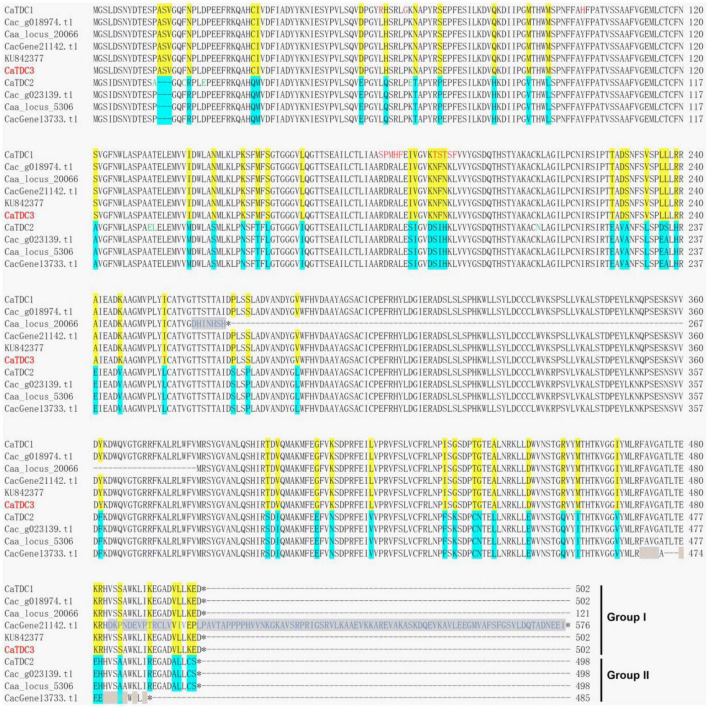
Multiple amino acid residue sequences alignment of the functionally characterized CaTDC1 and CaTDC2 and the annotated TDCs from *Camptotheca acuminata*. The amino acid residue sequences of CaTDC1 (López-Meyer and Nessler, 1997), CaTDC2 (López-Meyer and Nessler, 1997), and KU842377 (Sadre et al., 2016) were retrieved from NCBI database. Caa_locus_20066 and Caa_locus_5306 were retrieved from *C. acuminata* transcriptome dataset downloaded from http://medicinalplantgenomics.msu.edu by Góngora-Castillo et al. (2012). Cac_g018974.t1 and Cac_g023139.t1 were retrieved from *C. acuminata* genome dataset downloaded from http://medicinalplantgenomics.msu.edu by Zhao et al. (2017). CacGene21142.t1 and CacGene13733.t1 were retrieved from *C. acuminata* genome dataset downloaded from https://doi.org/10.6084/m9.figshare.12570599 by Kang et al. (2021). The different amino acid residues between Group I and Group II were highlighted in yellow and cyan, respectively. The red amino acid residues showed that they are different from others in Group I.

*Camptotheca acuminata*, a leading producer of the well-known plant-derived antitumor camptothecin (Figure 1B), had been reported to contain two autonomously regulated *TDC* genes, *CaTDC1* and *CaTDC2* (López-Meyer and Nessler, 1997). Both CaTDC1 and CaTDC2 were confirmed to exhibit decarboxylation activity toward L-tryptophan and the decarboxylation product tryptamine would be integrated into camptothecin (López-Meyer and Nessler, 1997). The subsequent transcriptome (Góngora-Castillo et al., 2012) and genome (Zhao et al., 2017; Kang et al., 2021) sequencing datasets suggested the presence of more *TDC*s in *C. acuminata* (Figure 2). Based on the multiple amino acid residue sequences alignment results, we herein cloned a distinct *TDC*, *CaTDC3* (GenBank: ON964510), from camptothecin-producing *C. acuminata*. Heterologous overexpression and functional characterization clarified the catalytic decarboxylation activity of CaTDC3 toward L-tryptophan. Homology modeling and molecular docking suggested CaTDC3’s potential catalytic promiscuity toward different substituted L-tryptophan analogs. Twenty-eight analogs, including hydroxylated, halogenated, and C^β^ -methylated L-tryptophans, 1-thio- and 7-aza-L-tryptophans, and an L-tryptophan’s degradation product, were employed to clarify the substrate scope of CaTDC3. The present works revealed that CaTDC3 exhibits strict stereoselectivity and catalytic promiscuity for L-tryptophan derivatives and analogs.

## Materials and methods

### Plant materials, total RNA extraction and isolation, and cDNA library construction

The *C. acuminata* seeds collection, seedlings growth, total RNA extraction and isolation, and cDNA library construction were performed on the basis of the reported procedures (Yang et al., 2019; Awadasseid et al., 2020).

### CaTDCs’ amino acid residue sequences analyses

The amino acid residue sequences of *CaTDC1* (GenBank: U73656) (López-Meyer and Nessler, 1997), *CaTDC2* (GenBank: U73657) (López-Meyer and Nessler, 1997), and a putative *CaTDC* (GenBank: KU842377) (Sadre et al., 2016) were retrieved from the National Center for Biotechnology Information (NCBI) database. The annotated TDCs Caa_locus_20066 and Caa_locus_5306 were retrieved from the *C. acuminata* transcriptome dataset downloaded from http://medicinalplantgenomics.msu.edu (Góngora-Castillo et al., 2012). The annotated TDCs Cac_g018974.t1 and Cac_g023139.t1 were retrieved from the *C. acuminata* genome dataset downloaded from http://medicinalplantgenomics.msu.edu by Zhao et al. (2017). The annotated TDCs CacGene21142.t1 and CacGene13733.t1 were retrieved from the *C. acuminata* genome dataset downloaded from https://doi.org/10.6084/m9.figshare.12570599 by Kang et al. (2021).

Multiple amino acid residue sequences alignment was performed by using Clustal Omega software.^1^ The results of the multiple amino acid residue sequences alignment of the aforementioned TDCs were presented in Figure 2.

### Open reading frame cloning of *CaTDC3*

According to the multiple amino acid residue sequences alignment results (Figure 2), the specific primers CaTDC3-F (ATGGGTAGCCTTGATTCCAATTACG, 5′→ 3′) and CaTDC3-R (TCAATCCTCTTTCAGGAGAACATCC, 5′→ 3′) were designed to clone the whole open reading frame of the target *TDC*. All primers were synthesized, purified, and authenticated by TsingKe Biological Technology (Chengdu) Co., Ltd. Using the previously prepared cDNA mixture (Yang et al., 2019; Awadasseid et al., 2020) as template and CaTDC3-F and -R as primers, the open reading frame of *CaTDC3* was obtained from the PCR amplification reaction by following the cycling conditions: 1 cycle of 95°C for 2 min and 35 cycles of 95°C for 20 s, 55°C for 20 s, 72°C for 1 min followed by a final extension at 72°C for 5 min. The amplification products were gel-purified and ligated into the pGM-T vector [Tiangen Biotech (Beijing) Co., Ltd, China]. The constructs were chemically transformed into *E. coli* DH5α competent cells. The following DNA sequencing was performed in both directions by TsingKe Biological Technology (Chengdu) Co., Ltd. The nucleotide sequence was analyzed by using the similarity search BLAST program.

### Bioinformatics analyses, homology modeling, and molecular modeling

The properties of CaTDC3 were estimated by using the online ExPASy ProtParam tools.^2^ To assess the evolutionary relationship between CaTDC3 and other TDC homologs from different plant species, CaTDC3 was set as query to search the NCBI database by using BLASTp searches and the amino acid residue sequences of the functionally characterized TDCs were retrieved from the NCBI database. The amino acid residue sequences of TDCs were aligned by using the Clustal W program.^3^ MEGA 7 software was employed to construct a phylogenetic tree with neighbor-joining method. Bootstrap analysis with 1,000 replicates was conducted to obtain the confidence levels for the branches.

The crystal structure of *C. roseus* TDC (CrTDC, PDB: 6EEW) (Torrens-Spence et al., 2020) was used as a template to build the three-dimension structure of CaTDC3 by using the SWISS-MODEL toolkit from ExPASy web server.^4^ Based on the predicted three-dimension structure, the structurally and functionally important regions of CaTDC3 were identified from the deduced amino acid residue sequence of CaTDC3 by using AutoDock 1.5.7.^5^ The number of points in *x*-, *y*-, and *z*-dimension are 50 and the spacing is 0.375. The *x*, *y*, and *z* center are −14.917, −8.839, and −20.646, respectively. Genetic algorithm was selected for the search parameters and the numbers of runs are 50. The genetic algorithm (4.2) was selected for output. The conformation 4 was selected for further analyses. Simulations with bonded L-tryptophan were performed with PyMOL (2.5.2) molecular graphics system.

### Heterologous overexpression and preparation of recombinant CaTDC3

For the heterologous overexpression of CaTDC3 in *E. coli* BL21(DE3), the specific primes CaTDC3-BN-F (CGGGATCCATGGGTAGCCTTGATT, 5′→ 3′) and CaTDC3-BN-R (ATAGTTTAGCGGCCGCTCAATCCTCTT TCAG, 5′→ 3′) were designed and synthesized to amplify the whole open reading frame of *CaTDC3* from the above cloned full-length cDNA of *CaTDC3* by using a TransStart^®^ FastPfu DNA polymerase [TransGen Biotech (Beijing) Co., Ltd., China] with the following cycling conditions: 1 cycle of 95°C for 1 min and 35 cycles of 95°C for 20 s, 62°C for 20 s, 72°C for 30 s followed by a final extension at 72°C for 10 min. The PCR amplification products were gel-purified, digested with *Bam*HI and *Not*I, and ligated into the pET28a vector that was digested with the same endonucleases to obtain the recombinant plasmid pET28a-CaTDC3. *E. coli* DH5α competent cells were chemically transformed with the expression construct pET28a-CaTDC3. The nucleotide sequence was sequenced and analyzed by following the procedures mentioned above.

The verified recombinant plasmid pET28a-CaTDC3 was chemically transformed into *E. coli* BL21(DE3) competent cells to generate the recombinant strain which was grown on Luria-Bertani (10 g L^–1^ of tryptone, 5 g L^–1^ of yeast extract, and 10 g L^–1^ of NaCl) plates supplemented with 50 μg ml^–1^ of kanamycin. A single colony of the recombinant strain was inoculated into 5 ml of Luria-Bertani broth containing kanamycin (50 μg ml^–1^) and the resulting mixture was incubated overnight at 37°C, 180 rpm in a shaking incubator. An aliquot (500 μl) was inoculated into 500 ml of Luria-Bertani broth supplemented with the same antibiotics and incubated at 37°C, 180 rpm. When the optical density (OD_600 *nm*_) of the culture reached 0.6–0.8, 1 mM isopropyl β-D-L-thiogalactopyranoside (Sangon Biotech, Shanghai, China) was added into the culture to induce the overexpression of recombinant CaTDC3. The resulting mixture was incubated at 16°C, 150 rpm for 18 hrs. The cells were harvested by centrifugation at 4°C, 4,000 rpm for 15 min. The cell pellets were washed twice by PBS buffer (20 mM NaH_2_PO_4_, 500 mM NaCl, 10% glycerol, 10 mM imidazole, 5 mM β-mercaptoethanol, pH 7.4) and re-suspended in the same buffer containing 1 mg L^–1^ of lysozyme and 1 mM phenylmethylsulfonyl fluoride. The resulting solution was sonicated in an ice-bath at a 10 s interval until the mixture was homogeneous. The supernatant soluble fraction was recovered by centrifugation at 4°C, 12,000 rpm for 30 min. The supernatant was filtered through a 0.45 μm PES membrane and the resulting filtrate was loaded onto a gravity column of nickel nitrilotriacetic acid resin (Ni-NTA, Sangon Biotech, Shanghai, China) pre-equilibrated with binding buffer (20 mM NaH_2_PO_4_, 500 mM NaCl, pH 7.4). The binding buffer with different concentration of imidazole (10, 50, and 250 mM) was employed to elute the column. Aliquots of each fraction were analyzed on a 12% SDS-PAGE. Fractions containing His_6_-tagged CaTDC3 were pooled, desalted, and concentrated by using an Amicon Ultra centrifugal filter MWCO 30 kDa (Merck Millipore Ltd., United States) with dialysis buffer (50 mM NaH_2_PO_4_, 150 mM NaCl, pH 7.6). The purified protein was stored in dialysis buffer containing 20% glycerol at −80°C for further usage. The purified protein samples were analyzed on 12% SDS-PAGE and the concentration was estimated by using the ε_280 *nm*_ = 84,060 M^–1^ cm^–1^ for CaTDC3 calculated from ExPASy ProtParam.

### Enzymatic activity assay and optimization for CaTDC3-catalyzed decarboxylation reaction

The catalytic activity of CaTDC3 was measured as described previously (Liu et al., 2012). Briefly, the reaction was performed in 100 μl of PBS buffer (50 mM, pH 7.6) containing 4 μM PLP, 5 mM L-tryptophan, and 16 μM CaTDC3. The reaction mixtures were incubated at 37°C for 5, 10, and 20 min. At each time point the mixture was quenched with 100 μL of chill methanol to precipitate the protein. All precipitates were removed by centrifugation at 12,000 rpm for 10 min. The resulting supernatant was subjected to analyze by high-performance liquid chromatography (HPLC)-diode array detector (DAD) that was equipped with a C_18_ column (250 mm × 4.6 mm, 5 μm, Altima). The mobile phase consisted of solvent A (CH_3_CN) and solvent B (H_2_O containing phosphoric acid, pH 2.3) followed a gradient elution program (0 min, 5% A; 16 min, 50% A; 18 min, 95% A; and 23 min, 95% A) at a flow rate of 1 ml min^–1^ at room temperature, monitored by a DAD at 280 nm.

All enzymatic reactions were performed in triplicate and each reaction was initiated by the addition of CaTDC3.

To determine the optimal pH value of the reaction buffer for CaTDC3-catalyzed decarboxylation reaction, the enzymatic reactions were performed in various buffer systems, including AcOH-NaOAc buffer (50 mM, pH 5.0), Na_2_HPO_4_-NaH_2_PO_4_ buffer (50 mM, pH 6.5–7.6), Tris–HCl buffer (50 mM, pH 8.0–8.5), and Glycine-NaOH buffer (50 mM, pH 9.0–10.0), at 37°C. The effects of the reaction temperature on the catalytic activity of CaTDC3 were evaluated by incubation of the enzymatic reaction mixtures at 15, 25, 32, 37, 42, 50, and 60°C, respectively, with a constant pH 7.6. Under the optimal reaction temperature and buffer, the time course experiments with varying enzyme concentrations demonstrated that CaTDC3 (0.16 μM) exhibits an initial linear reaction rate within 10 min.

### Kinetics parameters for CaTDC3-catalyzed decarboxylation reaction

The calibration curve for tryptamine was established to quantitate the formation of tryptamine by HPLC-DAD analyses. Briefly, the standard tryptamine solutions with 0, 0.5, 1, 2.5, 5, 10, 25, 50, and 100 μM were prepared respectively and subjected to HPLC-DAD analyses by using the methods described above. The calibration curve was made by following a linear fit for the relationship of the specific concentration of tryptamine versus the corresponding peak area integral at 280 nm. Limits of determination and quantification were determined as signal/noise = 3 and 10, respectively. The kinetics parameters of CaTDC3 were determined under the aforementioned optimal reaction conditions. The enzymatic reaction was performed in 100 μL of PBS buffer (50 mM NaH_2_PO_4_, pH 7.6) containing 4 μM PLP, 0.16 μM CaTDC3, and L-tryptophan with different concentrations (10, 25, 50, 100, 250, and 500 μM). The kinetic constants were calculated with non-linear regression analysis using Origin 9.0 software.

### Substrate scope of CaTDC3-catalyzed decarboxylation reaction

To investigate the substrate scope and selectivity of CaTDC3, 28 L-tryptophan derivatives and analogs were introduced as potential substrates under the optimal reaction conditions determined for CaTDC3-catalyzed L-tryptophan decarboxylation. The fluoro-, chloro-, bromo-, methyl-, methoxy-, and 7-aza-L-tryptophan analogs were purchased from Amatek Scientific, Suzhou, China. L-Tryptophan, 5-hydroxy-L-tryptophan, N^α^ -acetyl-L-tryptophan, and L-tryptophan methyl ester were purchased from Aladdin Industrial Corporation, Shanghai, China. 6-Hydroxy-L-tryptophan, 1-thio-L-tryptophan, 7-aza-tryptamine, 5-chloro-tryptamine hydrochloride, and 5-bromo-tryptamine hydrochloride were purchased from Bide Pharmatech Ltd., Shanghai, China. 5-Hydroxy-tryptamine was purchased from Macklin Biochemical Co., Ltd., Shanghai, China. 5-Fluoro-tryptamine hydrochloride was purchased from Titan scientific Co., Ltd., Shanghai, China. Kynuramine dihydrobromide was purchased from Sigma-Aldrich, American. The enzymatic and control reaction assays were conducted in duplicated. The HPLC-DAD analyses were performed on the basis of the aforementioned analytic methods.

### Characterization of the product from CaTDC3-catalyzed decarboxylation reaction

Generally the aforementioned L-tryptophan derivatives and analogs were employed as substrates in the CaTDC3-catalyzed decarboxylation reactions, the enzymatic products were identified by comparison of their HPLC retention time and UV profile with those of the authentic standards. Then the individual product in the enzymatic reaction was collected and characterized by high resolution mass spectrometry (HRMS) data analysis. The pooled product was directly analyzed on a Bruker microTOF-Q mass spectrometer (Bremen, Germany) equipped with an electrospray ionization interface (ESI). The HRMS(ESI) was operated in positive ion mode and the spectra were collected in the enhanced full mass scan mode from *m/z* 50–1,500.

For HPLC-DAD analysis of the enzymatic reaction with 7-aza-L-tryptophan as substrate, the mobile phase consisted of solvent A (CH_3_CN) and solvent B (H_2_O containing-phosphoric acid, pH 2.3) followed a gradient elution program (0 min, 5% A; 16 min, 50% A; 18 min, 95% A; and 23 min, 95% A) at a flow rate of 0.5 ml min^–1^, monitored by a DAD at 280 nm. When 5-hydroxy- and 6-hydroxy-L-tryptophans were used as substrate, the HPLC-DAD analyses were performed with the mobile phase consisted of solvent A (CH_3_OH) and solvent B (H_2_O containing-phosphoric acid, pH 2.3) followed a gradient elution program (0 min, 5% A; 20 min, 20% A; 23 min, 95% A; and 27 min, 95% A) at a flow rate of 1.0 ml min^–1^, monitored by a DAD at 280 nm. The enzymatic reaction with L-tryptophan methyl ester as substrate was analyzed by HPLC-DAD equipped with an Agilent ZORBAX SB C_18_ analytic column (250 mm × 4.6 mm, 5 μm). The mobile phase consisted of solvent A (CH_3_CN) and solvent B (H_2_O containing-phosphoric acid, pH 2.3) followed a gradient elution program (0 min, 5% A; 16 min, 50% A; 18 min, 95% A; and 20 min, 95% A) at a flow rate of 1.0 ml min^–1^, monitored by a DAD at 280 nm.

To characterize the structure of the product from the CaTDC3-catalyzed decarboxylation reaction using 1-thio-L-tryptophan as substrate, 2.2 mg of 1-thio-L-tryptophan was added into 2 ml of NaH_2_PO_4_-NaOH buffer (50 mM, pH 7.6) containing CaTDC3 (3 μM) and PLP (4 μM). The reaction was incubated at 37°C for 48 h. The reaction mixture was extracted with EtOAc (2 ml) three times. EtOAc was removed to give the crude product. HRMS(ESI) *m/z* 178.0698 [M + H]^+^; ^1^H NMR (400 MHz, CDCl_3)_ δ 8.03 (1H, dd, *J* = 7.0, 1.0 Hz), 7.92 (1H, dd, *J* = 7.3, 1.2 Hz), 7.56 (1H, td, *J* = 7.5, 1.2 Hz), 7.52 (1H, td, *J* = 7.5, 1.0 Hz), 7.32 (1H, s), 3.26 (2H, t, *J* = 6.3 Hz), and 3.18 (2H, t, *J* = 6.3 Hz).

## Results

### Multiple amino acid residue sequences analyses of CaTDCs

Multiple amino acid residue sequences alignment of the functionally characterized CaTDCs, i.e., CaTDC1 and CaTDC2 (López-Meyer and Nessler, 1997), a putative *CaTDC* (GenBank: KU842377) (Sadre et al., 2016), and the annotated CaTDCs from the *C. acuminata* transcriptome (Góngora-Castillo et al., 2012) and genome (Zhao et al., 2017; Kang et al., 2021) datasets showed these TDCs could be classified into two groups in view of more than 60 amino acid residues difference highlighted in yellow for Group I and in cyan for Group II, respectively (Figure 2). CaTDC1, Caa_locus_20066, Cac_g018974.t1, CacGene21142.t1, and KU842377 were classified into Group I, while CaTDC2, Caa_locus_5306, Cac_g023139.t1, CacGene13733.t1 were grouped in Group II (Figure 2). In Group I, Cac_g018974.t1 and KU842377 showed identical amino acid residue sequences (Figure 2). Caa_locus_20066 displayed 7 different amino acid residues at 261-267, while CacGene21142.t1 had a different C-terminus at 484-576 (Figure 2). Closest analyses of their amino acid residue sequences in Group I demonstrated that the aforementioned four TDCs must be the same TDC that has 13 amino acid residues (in red color) different from CaTDC1 (Figure 2). CaTDC2 has 6 different amino acid residues (in green color), comparing with others in Group II (Figure 2).

### Molecular cloning and bioinformatics analyses of CaTDC3

The above mentioned multiple amino acid residue sequences alignment of the functionally characterized CaTDCs and the annotated TDCs from *C. acuminata* transcriptome and genome datasets implied the presence of 13 different amino acid residues in Group I CaTDCs (Figure 2). To further verify the presence of different amino acid residues and clarify the catalytic function of CaTDC in Group I, we cloned a TDC-encoding gene from *C. acuminata* on the basis of the aforementioned alignment results (Figure 2). The cloned gene is an open reading frame of 1,509 bp, encoding a TDC with 502-amino acid residues that is identical to those of KU842377. It was designated *CaTDC3* (GenBank: ON964510) in view of 13 and 66 different amino acid residues, respectively, from functionally characterized CaTDC1 and CaTDC2 (Figure 2).

**FIGURE 3:**
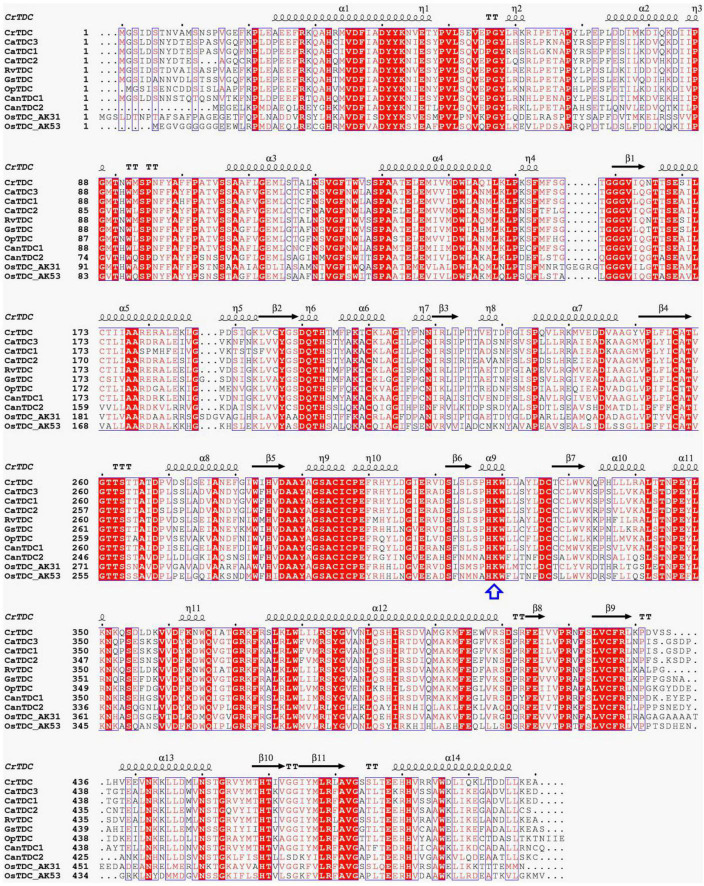
Multiple amino acid residue sequences alignment of biochemically characterized plant TDCs. The key lysine involved in interaction with PLP was highlighted with a blue arrow. TDCs are from *Catharanthus roseus* (CrTDC, AAA33109.1), *Rauvolfia verticillata* (RvTDC, ADL28270.1), *Gelsemium sempervirens* (GsTDC, AXK92562.1), *Ophiorrhiza pumila* (OpTDC, BAC41515.1), *Capsicum annuum* (CanTDC1, ACN62127.1; CanTDC2, ACN62126.1), *Oryza sativa* Japonica Group (OsTDC_AK31, BAG91223.1; OsTDC_AK53, BAG95977.1), and *Camptotheca acuminata* (CaTDC1, AAB39708.1; CaTDC2, AAB39709.1). The results were graphed by using ESPript 3.0.

**FIGURE 4:**
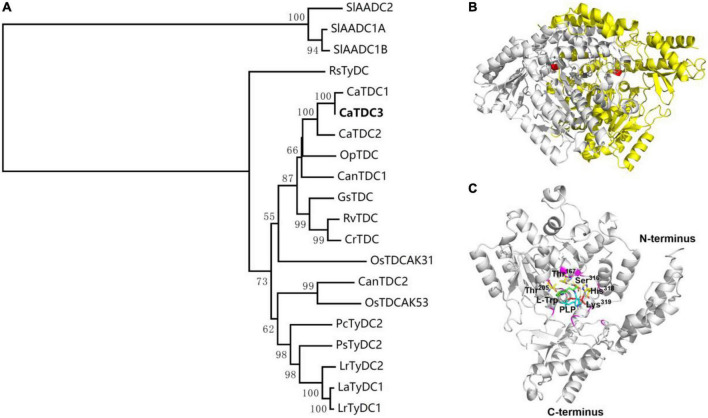
Phylogenetic relationship of CaTDC3 with plant aromatic amino acid decarboxylases **(A)**, predicted three-dimension structure of dimeric CaTDC3 **(B)**, and schematic key active region of CaTDC3 **(C)**. The amino acid residue sequences of plant aromatic amino acid decarboxylases (AADCs) were obtained by using the NCBI search engine. The phylogenetic tree was constructed by MEGA 7.0 software package and neighbor-joining program with 1,000 boots trapped value support and a Poisson correction. The bootstrap values higher than 50% are given at the nodes. In the homodimer structure of CaTDC3, one was colored in gray and another was in yellow. The key Lys319 was marked in red. The amino acid residues involved in interactions with L-tryptophan are Thr167, Thr205, Ser316, His318, and Lys319. PLP was in cyan.

CaTDC3 was predicted to have a calculated molecular mass of 55.6 kDa and an isoelectric point of 6.56. Similarity search showed that CaTDC3 shares 54–97% amino acid residue identities with the functionally characterized plant TDCs (Figure 3). Based on the catalytic mechanisms of plant TDCs (López-Meyer and Nessler, 1997; Liu et al., 2012), Lys319 is most likely to be the key catalytic amino acid in CaTDC3 (Figure 3). The phylogenetic tree analysis showed that the plant aromatic amino acid decarboxylases should be grouped into three groups depending on the substrate specificity for tryptophan, tyrosine, and phenylalanine, respectively (Figure 4A). CaTDC3 falls into the clade of TDCs in which TDCs from *C. roseus* (CrTDC) (De Luca et al., 1989), *R. verticillata* (RvTDC) (Liu et al., 2012), *Gelsemium sempervirens* (GsTDC) (Franke et al., 2019), and *O. pumila* (OpTDC) (Yamazaki et al., 2003) had been functionally characterized to be involved in the biosynthesis of monoterpene indole alkaloids, indicating CaTDC3 has similar catalytic function (Figure 4A).

The crystal structure of CrTDC with 2.05 angstrom resolution (Torrens-Spence et al., 2020) was employed to build the three-dimension structure of CaTDC3. As depicted in Figure 4B, CaTDC3 is present in a dimeric form, in which the catalytically active domain consisted of Thr205, Thr167, Ser316, His318, and Lys319 (Figure 4C).

### Heterologous overexpression and catalytic properties of CaTDC3

**FIGURE 5:**
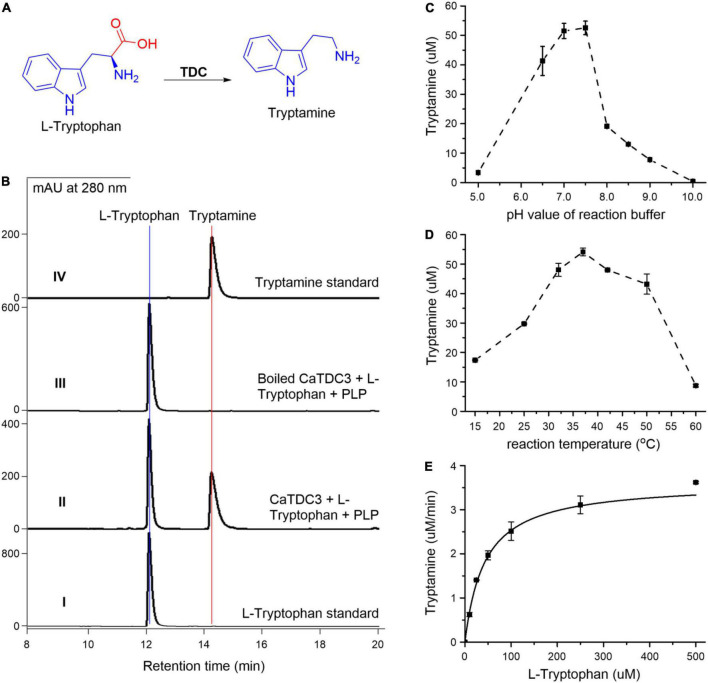
Functional and biochemical characterization of CaTDC3. CaTDC3-catalyzed decarboxylation reaction of L-tryptophan **(A)**. **(B)** HPLC-DAD traces of the decarboxylation reaction mixture catalyzed by CaTDC3 (panel II) and boiled CaTDC3 (panel III). Panels I and IV are authentic L-tryptophan and standard tryptamine, respectively. The effects of pH value of reaction buffers **(C)** and reaction temperature **(D)** on the catalytic activity of CaTDC3. **(E)** The steady kinetics of CaTDC3 toward L-tryptophan.

To clarify the catalytic decarboxylation function of CaTDC3 (Figure 5A), the entire open reading frame of *CaTDC3* was subcloned into a pET28a vector to generate an N-terminal His_6_-tagged expression construct. The intact CaTDC3 was heterologously overexpressed in *E. coli* BL21(DE3) and the recombinant CaTDC3 was purified to homogeneity (Supplementary Figure 1) by using Ni-NTA affinity chromatograph.

The enzymatic decarboxylation activity assays were conducted by incubation of L-tryptophan, PLP, and CaTDC3. HPLC-DAD analyses showed the presence of an enzymatic reaction product in the CaTDC3-catalyzed reaction mixture (Figure 5B, panel II), compared with the control experiment using the boiled CaTDC3 as catalyst (Figure 5B, panel III). The aforementioned product was demonstrated to have identical HPLC retention time (Figure 5B, panel II) and UV profile (Supplementary Figure 2A, panel I) to those of the authentic tryptamine (Figure 5B, panel IV and Supplementary Figure 2A, panel II). The protonated molecule at *m/z* 161.1082 ([M + H]^+^) in the HRMS(ESI) of the enzymatic product (Supplementary Figure 2B) confirmed that the CaTDC3-catalyzed reaction product is tryptamine, the decarboxylation product of L-tryptophan.

The recombinant CaTDC3 exhibited a maximum catalytic activity when the reaction was performed in the reaction buffer with pH = 7.6 (Figure 5C) and the reaction temperature was set at 37°C (Figure 5D). Under the aforementioned optimal reaction conditions, CaTDC3 showed an apparent *K*_*m*_ value of 48 ± 2 μM and *V*_*max*_ value of 4.10 ± 0.07 μM min^–1^ for L-tryptophan (Figure 5E).

### Molecular docking of CaTDC3 with L-tryptophan

**FIGURE 6:**
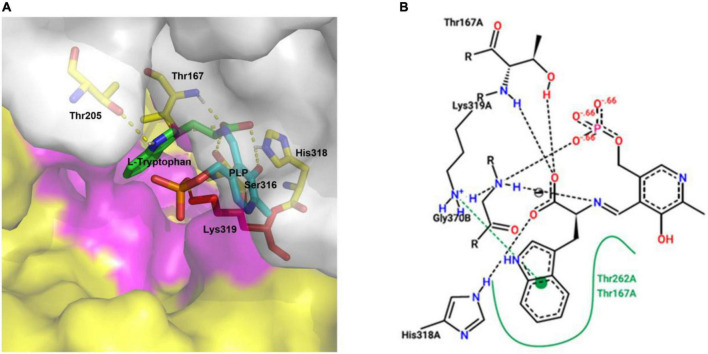
Molecular docking of CaTDC3 with L-tryptophan **(A)** and its concise diagram **(B)**. The catalytically active pocket of CaTDC3 was composed of two monomers, one in yellow and another in light gray, respectively. The amino acid residues Thr167, Thr205, Ser316, His318, and Lys319 were highlighted. The other amino acid residues within 5 angstrom around L-tryptophan were shown in purple. L-Tryptophan, PLP, and the key catalytic amino acid Lys319 were presented in green, cyan, and red, respectively. The binding energy was calculated to be –5.91 kcal/mol.

The predicted three-dimension structure of CaTDC3 (Figures 4B,C) was employed to perform molecular docking using L-tryptophan as substrate (Figure 6A). The catalytically active pocket of CaTDC3 was composed of amino acid residues from two monomers, one in yellow and another in light gray (Figure 6A). The molecular docking results implied that L-tryptophan analogs with the substituent groups at indole ring and the side chain might be recognized by CaTDC3 in view of a relative big spatial volume of the catalytic pocket (Figures 6A,B). Thus, CaTDC3 may catalyze the decarboxylation of L-tryptophan analogs.

### Enzymatic promiscuity of CaTDC3

Plant TDCs are members of aromatic amino acid decarboxylases family (Figure 4A), which implied that some TDCs might recognize other aromatic amino acids and catalyze the corresponding decarboxylation reaction. Herein, various L-tryptophan derivatives and analogs were employed to investigate the catalytic promiscuity of CaTDC3 toward these substrates.

#### Basic structure requirements of substrate for CaTDC3-catalyzed decarboxylation reaction

**FIGURE 7:**
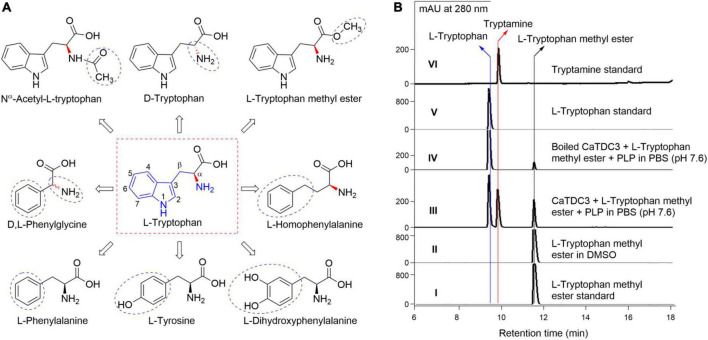
Basic structure requirements of substrate for CaTDC3-catalyzed decarboxylation reaction. Chemical structures of L-tryptophan analogs tested in the present work **(A)**. **(B)** HPLC-DAD analyses of the decarboxylation reaction in PBS buffer catalyzed by CaTDC3 (panel III) and boiled CaTDC3 (panel IV), respectively. Panels I, V, and VI are the HPLC-DAD traces of authentic L-tryptophan methyl ester, L-tryptophan, and tryptamine, respectively. Panel II is the HPLC-DAD trace of L-tryptophan methyl ester dissolved in DMSO. The blue cycles indicated the groups different from that of L-tryptophan.

Firstly, D-tryptophan (Figure 7A) was used as a possible substrate for the CaTDC3-catalyzed decarboxylation reaction. The results (Supplementary Figure 3A) showed that D-tryptophan cannot be decarboxylated by CaTDC3 and CaTDC3 exhibit a strict stereoselectivity for L-tryptophan.

The amino group (-NH_2_) of L-tryptophan should not be blocked for the CaTDC3-catalyzed decarboxylation reaction in view of that there is not any related product was observed when N^α^ -acetyl-L-tryptophan (Figure 7A) was introduced as substrate (Supplementary Figure 3B).

L-Tryptophan methyl ester (Figure 7A) was introduced to test the importance of the free carboxyl group (-COOH). The decarboxylation product tryptamine was detected in the CaTDC3-catalyzed decarboxylation reaction using L-tryptophan methyl ester as substrate (Figure 7B, panel III), compared with the control experimental (Figure 7B, panel IV). Carefully checking showed that L-tryptophan was detected after L-tryptophan methyl ester was added into the reaction buffer and most of L-tryptophan methyl ester disappeared while a large amount of L-tryptophan was detected in the control experimental (Figure 7B, panel IV). With the prolongation of the reaction time, both L-tryptophan and tryptamine were detected and their contents were found to be increased gradually (Figure 7B, panel III). There was not any product detected when L-tryptophan methyl ester was dissolved in DMSO only (Figure 7, panel II). The results indicated that L-tryptophan methyl ester was hydrolyzed to form L-tryptophan spontaneously in PBS reaction buffer (pH 7.6) and the latter one was decarboxylated by CaTDC3.

Five L-tryptophan analogs with different substituted aromatic rings and side chains, including L-tyrosine, L-phenylalanine, L-DOPA (3,4-dihydroxyphenylalanine), L-homophenylalanine, and D,L-phenylglycine (Figure 7A), were employed to evaluate the substrate structure requirements under the aforementioned optimal reaction conditions for CaTDC3-catalyzed decarboxylation reaction. None of them could be decarboxylated by the catalysis of CaTDC3 (Supplementary Figures 3C–G), which revealed that both the indole ring and the side chain are very important for the potential substrates of the CaTDC3-catalyzed decarboxylation reaction.

### The effects of the substituent groups at the indole ring of L-tryptophan on CaTDC3-catalyzed decarboxylation reaction

**FIGURE 8:**
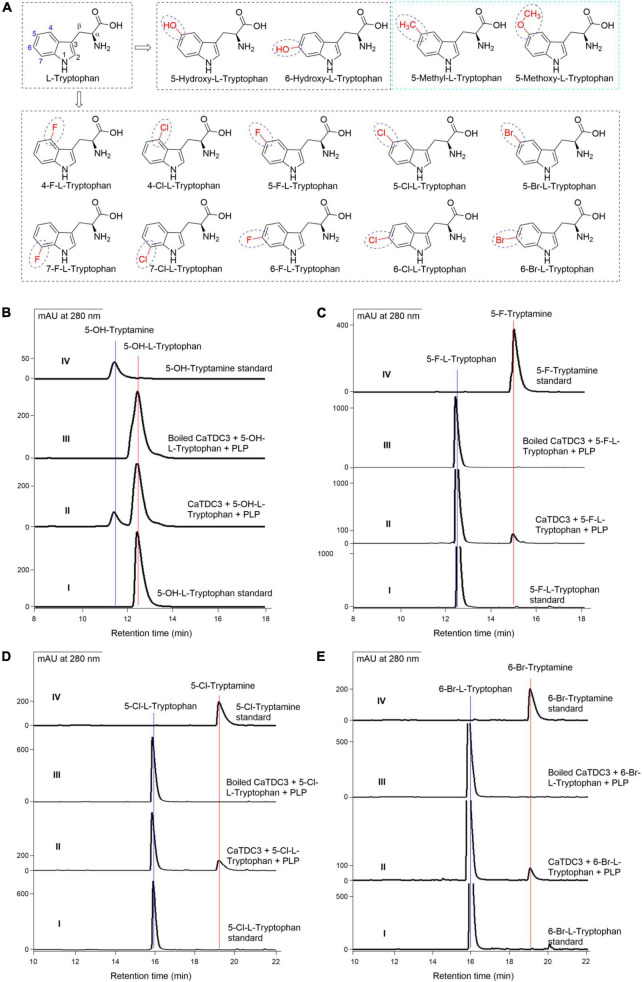
The effects of substituent groups at the indole ring of L-tryptophan on CaTDC3’s decarboxylative activity. Chemical structure of L-tryptophan analogs with diverse substituent groups at the indole ring tested in the present work **(A)**. **(B)** HPLC-DAD analyses of authentic 5-hydroxy-L-tryptophan (panel I), the enzymatic reaction mixture with CaTDC3 (panel II), and boiled CaTDC3 (panel III) as catalyst, respectively, and the standard 5-hydroxytryptamine (panel IV). **(C)** HPLC-DAD analyses of the standard 5-fluoro-L-tryptophan (panel I), the enzymatic reaction mixture with CaTDC3 (panel II), and boiled CaTDC3 (panel III) as catalyst, respectively, and the standard 5-fluorotryptamine (panel IV). **(D)** HPLC-DAD analyses of the standard 5-chloro-L-tryptophan (panel I), the enzymatic reaction mixture with CaTDC3 (panel II), and boiled CaTDC3 (panel III) as catalyst, respectively, and the standard 5-chlorotryptamine (panel IV). **(E)** HPLC-DAD analyses of the standard 6-bromo-L-tryptophan (panel I), the enzymatic reaction mixture with CaTDC3 (panel II), and boiled CaTDC3 (panel III) as catalyst, respectively, and the standard 6-bromotryptamine (panel IV).

Carefully checking the three-dimension structure of CaTDC3 docked with L-tryptophan and PLP suggested that the substitution groups located at the aromatic ring and the side-chain might be recognized by CaTDC3 (Figure 6). The hydroxylated analog 5-hydroxy-L-tryptophan (Figure 8A) was employed to testify the aforementioned hypothesis. The HPLC-DAD analyses indicated that CaTDC3 can recognize 5-hydroxy-L-tryptophan and afford a product with retention time at 11.61 min (Figure 8B, panel II), comparing with the control experimental (Figure 8B, panel III). The product possesses an identical retention time (Figure 8B, panel II) and a UV profile (Supplementary Figure 4A, panel I) with those of 5-hydroxytryptamine (Figure 8B, panel IV and Supplementary Figure 4A, panel II). The enzymatic product showed the protonated molecule at *m/z* 177.1026 ([M + H]^+^) in the HRMS(ESI) (Supplementary Figure 4B), which confirmed that the CaTDC3-catalyzed reaction product is 5-hydroxy-tryptamine, the decarboxylation product of 5-hydroxy-L-tryptophan.

6-Hydroxy-L-tryptophan (Figure 8A) was verified to be recognized by CaTDC3 and the decarboxylation product, i.e., 6-hydroxy-tryptamine, was detected in the reaction mixture (Supplementary Figure 5).

Encouraged by the above results, we employed 10 halogenated L-tryptophans, including 4- F-, 5- F-, 6- F-, 7- F-, 4- Cl-, 5- Cl-, 6- Cl-, 7- Cl-, 5- Br-, and 6-Br-L-tryptophans (Figure 8A), to investigate the substrate scope of CaTDC3-catalyzed decarboxylation reaction. The corresponding decarboxylated products, i.e., halogenated tryptamines, were confirmed by HPLC-DAD and HRMS(ESI) data analyses from the reaction mixtures (Figures 8C–E and Supplementary Figures 6–15).

Two L-tryptophan analogs with larger substituent groups at C-5, i.e., 5-methyl- and 5-methoxy-L-tryptophans (Figure 8A), were used as substrate, respectively, in the CaTDC3-catalyzed decarboxylation reactions. However, the corresponding decarboxylation products could not be detected in the reaction mixture (Supplementary Figure 16).

#### The effects of the substituent groups at the side chain of L-tryptophan on CaTDC3-catalyzed decarboxylation reaction

**FIGURE 9:**
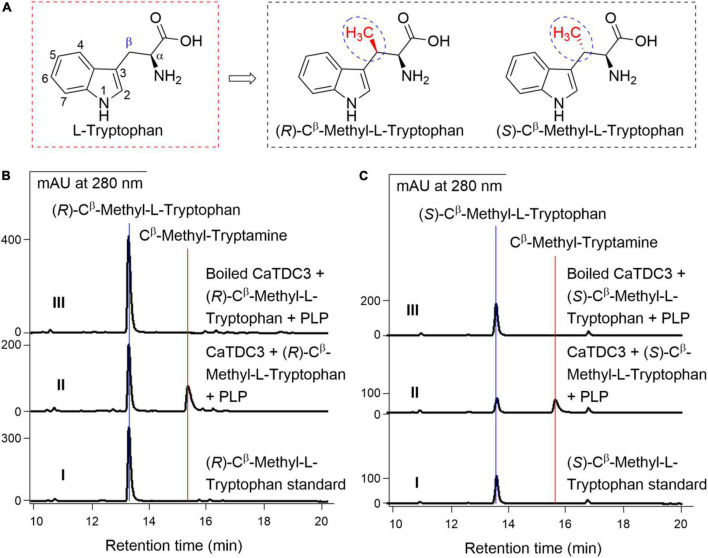
The effects of substituent groups at C^β^ of L-tryptophan on CaTDC3’s decarboxylative activity. Chemical structure of (*R*)-C^β^ -methyl- and (*S*)-C^β^ -methyl-L-tryptophans tested in the present work **(A)**. **(B)** HPLC-DAD analyses of the standard (*R*)-C^β^ -methyl-L-tryptophan (panel I), the enzymatic reaction mixture with CaTDC3 (panel II), and boiled CaTDC3 (panel III) as catalyst, respectively. **(C)** HPLC-DAD analyses of the standard (*S*)-C^β^ -methyl-L-tryptophan (panel I), the enzymatic reaction mixture with CaTDC3 (panel II), and boiled CaTDC3 (*panel* II) as catalyst, respectively.

For the CaTDC3-catalyzed decarboxylation reaction, the above experimental revealed that the carboxyl and amino groups of the side chain of L-tryptophan should not be modified (Figures 7A,B and Supplementary Figure 3B). Herein C^β^ -methyl-L-tryptophans (Figure 9A) were introduced to clarify the effects of the substituent group at the side chain of L-tryptophan on the CaTDC3-catalyzed decarboxylation reaction. Both (*R*)-C^β^ -methyl- and (*S*)-C^β^ -methyl-L- tryptophans could be recognized by CaTDC3 and the corresponding decarboxylation reaction product was detected (Figures 9B,C and Supplementary Figure 17).

#### The effects of the –NH group of indole ring of L-tryptophan on CaTDC3-catalyzed decarboxylation reaction

**FIGURE 10:**
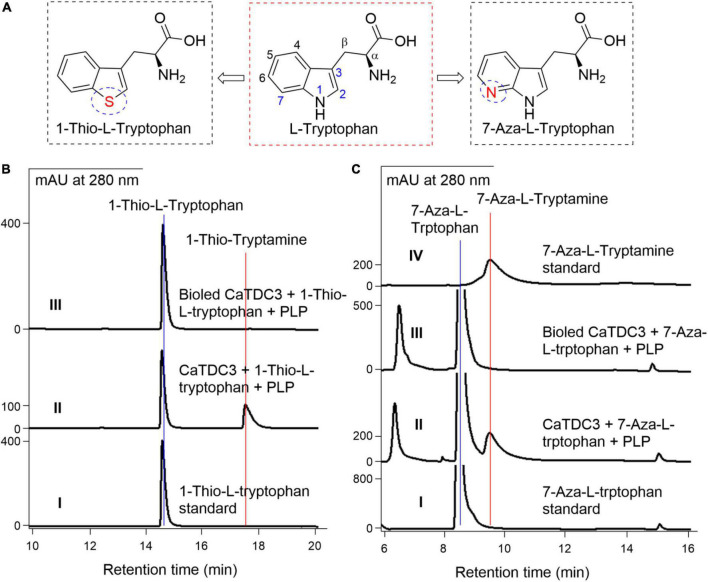
The effects of –NH displacement and aza-aromatic ring of L-tryptophan on CaTDC3’s decarboxylative activity. The substrate with distinct chemical structures from L-tryptophan tested in present work **(A)**. **(B)** HPLC-DAD analyses of the standard 1-thio-L-tryptophan (panel I), the enzymatic reaction mixture with CaTDC3 (panel II), and boiled CaTDC3 (panel III) as catalyst, respectively. **(C)** HPLC-DAD analyses of the standard 7-aza-L-tryptophan (panel I), the enzymatic reaction mixture with CaTDC3 (panel II), and boiled CaTDC3 (panel III) as catalyst, respectively, and the standard 7-aza-tryptamine (panel IV).

1-Thio-L-tryptophan (Figure 10A), the S analogs of the –NH group in the indole ring of L-tryptophan, could be recognized by CaTDC3 and its enzymatic reaction afforded a product (Figure 10B, panel II). The HRMS(ESI) of the enzymatic product showed the protonated molecule at *m/z* 178.0698 ([M + H]^+^) (Supplementary Figure 18) that is in good accordance to that of 1-thio-tryptamine. The ^1^H-NMR spectroscopic data (Supplementary Figures 19–21) of the enzymatic product were in perfect consistency with those reported for 1-thio-tryptamine (Szostak et al., 2014).

#### The effects of the N displacement of the carbon on phenyl ring of L-tryptophan on CaTDC3-catalyzed decarboxylation reaction

7-Aza-L-tryptophan (Figure 10A), a nitrogenous analog generated from the replacement of C-7 in L-tryptophan by an N atom, was confirmed to be converted into the corresponding tryptamine by CaTDC3 (Figure 10C). The enzymatic product was characterized as 7-aza-tryptamine by comparison of its HPLC-DAD (Figure 10C, panel II), UV (Supplementary Figure 22A, panel I), and HRMS(ESI) (Supplementary Figure 22B) data with those of the authentic standard.

#### The effects of the consistency of the indole ring of L-tryptophan on CaTDC3-catalyzed decarboxylation reaction

**FIGURE 11:**
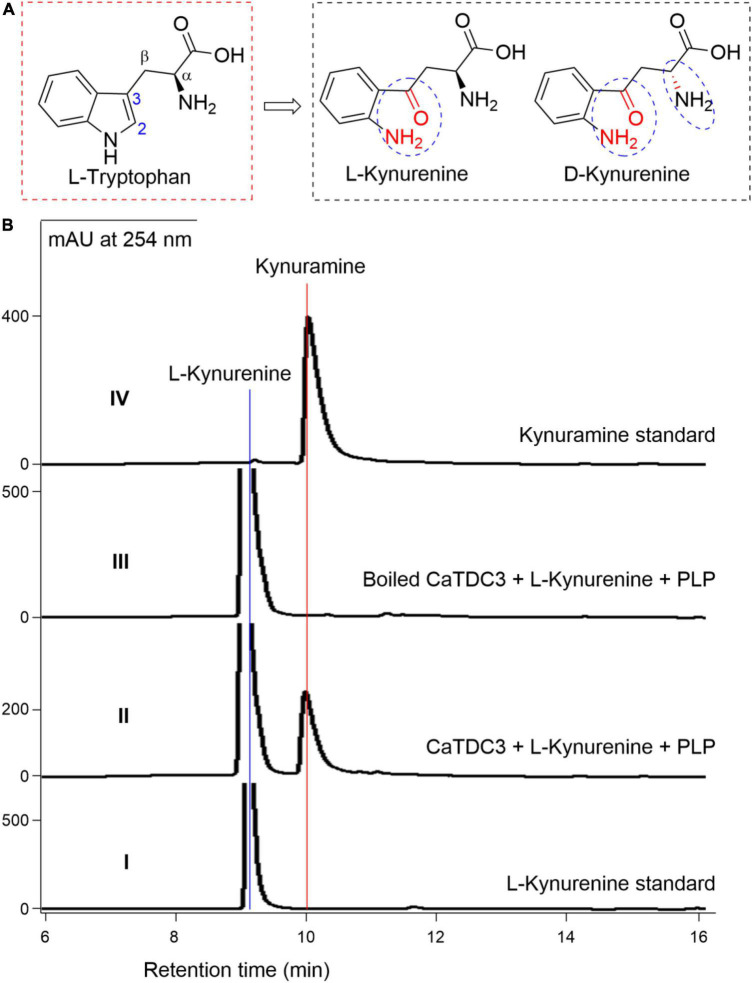
CaTDC3-catalyzed decarboxylation toward L-kynurenine. The chemical structure similarity of L-tryptophan and kynurenines tested in the present work **(A)**. **(B)** HPLC-DAD analyses the standard L-kynurenine (panel I), the enzymatic reaction mixture with CaTDC3 (panel II), and boiled CaTDC3 (panel III) as catalyst, respectively, and the standard kynuramine (panel IV).

To investigate the effects of the consistency of the indole ring of L-tryptophan on the CaTDC3-catalyzed decarboxylation reaction, L-kynurenine (Figure 11A) was employed as substrate. Comparison of the HPLC-DAD (Figure 11B), UV (Supplementary Figure 23A), and HRMS(ESI) (Supplementary Figure 23B) data with those of the authentic standard confirmed that CaTDC3 can recognize the unnatural amino acid and convert it into the related decarboxylation product, kynuramine. D-Kynurenine (Figure 11A) could not be accepted by CaTDC3 and no reaction product could be detected in the reaction mixture (Supplementary Figure 24).

## Discussion

Aromatic amines such as tryptamine, dopamine (3,4-dihydroxyphenylethylamine), and tyramine are fundamental biogenic amines in all living organisms (Facchini et al., 2000). These aromatic amines are converted from aromatic amino acids catalyzed by ubiquitous aromatic amino acid decarboxylases (AADCs), an ancient group of pyridoxal 5′-phosphate (PLP)-dependent enzymes (Facchini et al., 2000). AADCs share extensive amino acid residue identity, similar subunit structure, and kinetic properties (Facchini et al., 2000). However, these enzymes show striking difference in the substrate specificities (Facchini et al., 2000). For instance, L-DOPA decarboxylase, an animal AADC, is responsible for the synthesis of neurotransmitters dopamine and serotonin through decarboxylation of L-DOPA and 5-hydroxy-L-tryptophan, respectively (Franke et al., 2019). For plant AADCs, they have evolved with variations in activity and substrate specificity (Torrens-Spence et al., 2014; Günther et al., 2019). Plant tyrosine decarboxylases display decarboxylation activity toward L-tyrosine and L-DOPA, while TDCs catalyze decarboxylation of L-tryptophan and 5-hydroxy-L-tryptophan (Torrens-Spence et al., 2014). These plant-derived biogenic amines had been clarified to be integrated into diverse metabolic pathways for the synthesis of benzylisoquinoline and monoterpene indole alkaloids (Torrens-Spence et al., 2014, 2020). Additionally, one or more AADC-encoding genes had been cloned and functionally characterized from one plant species (Yamazaki et al., 2003; Kang et al., 2007; Park et al., 2009; Liu et al., 2012), which might be the expansion of gene families stemmed from an independent whole-genome duplication event during plant’s evolution (Kang et al., 2021).

### *CaTDC3* is a distinct tryptophan decarboxylase-encoding gene in camptothecin-producing *Camptotheca acuminata*

Two TDC-encoding genes, *CaTDC1* and *CaTDC2* (López-Meyer and Nessler, 1997), had been cloned from *C. acuminata*, a camptothecin-producing plant. They share 81% nucleotide sequence identity and 84% amino acid residue identity (López-Meyer and Nessler, 1997). *CaTDC1* was developmentally expressed at different levels in all tissues of *C. acuminata* and it was clarified to provide tryptamine for biosynthesis of camptothein under normal growth conditions (López-Meyer and Nessler, 1997). *CaTDC2* was specifically induced when *C. acuminata* plant tissues were treated with elicitors (López-Meyer and Nessler, 1997). The previous DNA gel blot analysis had implied a third *TDC* when *C. acuminata* DNA was digested with *EcoR* I (López-Meyer and Nessler, 1997). Herein, we mined and cloned *CaTDC3*, a TDC-encoding gene from *C. acuminata*. CaTDC3 shares 97 and 85% amino acid residue identities with CaTDC1 and CaTDC2, respectively (Figures 1, 2). It is in good accordance with previous transcriptome and genome sequencing results (Góngora-Castillo et al., 2012; Zhao et al., 2017; Kang et al., 2021) and DNA gel blot analysis (López-Meyer and Nessler, 1997).

### CaTDC3 displays characteristics of tryptophan decarboxylases

Recombinant CaTDC3 was clarified to catalyze an efficient decarboxylation conversion of L-tryptophan into tryptamine (Figure 5). The apparent *K*_*m*_ and *k*_*cat*_ values of recombinant CaTDC3 are 48 μM and 25.6 min^–1^, respectively, for L-tryptophan (Figure 5). CaTDC3’s *K*_*m*_ is closest to that of CrTDC (51.7 μM), whereas the *k*_*cat*_ value for CaTDC3 is higher than that of CrTDC (*k*_*cat*_ 5.1 min^–1^) (Runguphan et al., 2010). OpTDC (0.72 mM) (Yamazaki et al., 2003), OsTDC_*AK*31_ (0.69 mM) (Kang et al., 2007), and RvTDC (2.89 mM) (Liu et al., 2012) showed higher *K*_*m*_ values than that of CaTDC3. Taken together, CaTDC3 has greater efficiency in decarboxylation for L-tryptophan than other plant TDCs.

Multiple amino acid residue sequences alignment results showed that CaTDC1, CaTDC2, and CaTDC3 contain same conserve amino acid residues, identical catalytic amino acid residues, and similar three-dimensional structure, comparing with those of CrTDC (Figure 3). Phylogenetic tree analyses revealed that CaTDC1, CaTDC2, and CaTDC3 are very close to each other (Figure 4A). These results implied that they might display similar catalytic activity and substrate scope.

### CaTDC3 shows strict stereoselectivity for L-tryptophan and extensive substrate scope

CaTDC3 shows strict stereoselectivity for substrate in view of that only L-tryptophan could be recognized by CaTDC3 and afford the corresponding decarboxylative product (Figure 7). The conclusion was further supported by the following CaTDC3-catalyzed decarboxylation reactions using hydroxylated, halogenated, and C^β^ -methylated L-tryptophans as substrates and the related decarboxylation products were detected (Figures 8–10). Additionally, CaTDC3 could recognized L-kynurenine and catalyze its decarboxylation to generate kynuramine (Figure 11). While D-kynurenine could not be accepted by CaTDC3 (Supplementary Figure 24).

Homology modeling and molecular docking (Figure 6) revealed a relative big spatial volume of the catalytic pocket presented in CaTDC3, which implied that CaTDC3 might recognize the L-tryptophan analogs with the appropriate substituent groups at the indole ring and the side chain on the basis of the substrate basic requirement experimental results (Figure 7).

**FIGURE 12:**
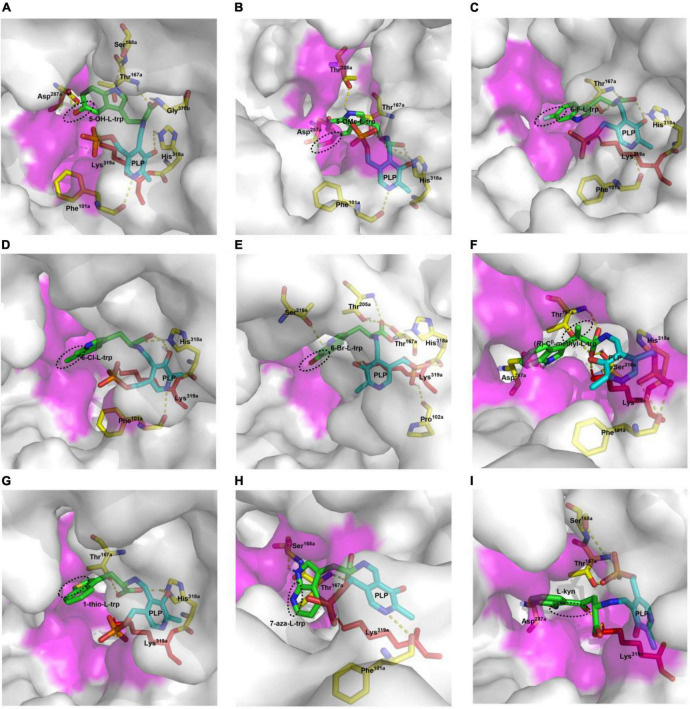
Molecular docking of CaTDC3 with different L-tryptophan analogs. The docked substrates are 5-hydroxy-L-tryptophan **(A)**, 5-methoxy-L-tryptophan **(B)**, 6-fluoro-L-tryptophan **(C)**, 6-chloro-L-tryptophan **(D)**, 6-bromo-L-tryptophan **(E)**, (*R*)-C^β^ -methyl-L-tryptophan **(F)**, 1-thio-L-tryptophan **(G)**, 7-aza-L-tryptophan **(H)**, and L-kynurenine **(I)**. The amino acid residues from one monomer to form the catalytically active domain were highlighted as Phe^101a^, Thr^167a^, Ser^168a^, Asp^287a^, His^318a^, and Lys^319a^. The other amino acid residues within 5 angstrom around L-tryptophan from another monomer were shown in purple.

Twelve hydroxylated and halogenated L-tryptophans (Figure 8) can be converted into the corresponding tryptamines by the catalysis of CaTDC3. However, L-tryptophan with larger groups substituted at C-5, i.e., 5-methyl- and 5-methoxy-L-tryptophan, cannot be converted by CaTDC3 (Figure 8). The molecular docking of CaTDC3 with 5-methoxy-L-tryptophan (Figure 12B) gave higher binding energy than that of CaTDC3 with 5-hydroxy-L-tryptophan (Figure 12A), indicating larger substituent groups at the indole ring could not be accepted.

The integration of halogen is a frequent modification for specialized metabolites and the resulting halogenated products display a wide range of biological activities and well pharmacological properties, including anticancer and antibiotic properties (Neumann et al., 2008; Runguphan et al., 2010). For instance, chlorinated alkaloids were generated from introduction of chlorine into tryptophan by halogenase and the following integration into monoterpene indole alkaloids (Runguphan et al., 2010). The halogenated tryptamines such as ^18^F-tryptamine, a potential radiopharmaceutical for monitoring of abnormal brain states occurring in diverse diseases, are extremely useful in nuclear medicine (Dragulska and Kańska, 2014). Here we showed that all halogenated L-tryptophans could be converted into halogenated tryptamine by CaTDC3 (Figure 8), indicating its potential usage in metabolic engineering for pharmaceutically important metabolites. The representative molecular docking of CaTDC3 with 6-F-L-tryptophan (Figure 12C), 6-Cl-L-tryptophan (Figure 12D), and 6-Br-L-tryptophan (Figure 12E), respectively, supported the above mentioned experimental results.

Molecular docking of CaTDC3 with C^β^ -methyl-L-tryptophan (Figure 12F) suggested the binding energy is −6.86 kcal mol^–1^ and CaTDC3 may catalyze decarboxylation of the aforementioned L-tryptophan analogs. The experimental results (Figure 9) revealed that both (*R*)- and (*S*)-C^β^ -methyl-L-tryptophans were decarboxylated by CaTDC3 efficiently.

1-Thio-L-tryptophan and 7-aza-L-tryptophan, two unnatural L-tryptophans substituted by hetero atoms, could be recognized by CaTDC3 and the related decarboxylation products were obtained successfully (Figure 10). Molecular docking of CaTDC3 with 1-thio-L-tryptophan (Figure 12G) and 7-aza-L-tryptophan (Figure 12H), respectively, suggested the binding energy are −6.53 and −7.51 kcal mol^–1^. Thus, the NH group of the indole ring and the C atom on the aromatic ring of L-tryptophan might be replaced by appropriate hetero atoms and the corresponding hetero-tryptamines could be obtained from CaTDC3-catalyzed decarboxylation reactions.

CaTDC3 cannot catalyze the decarboxylation of L-phenylalanine, L-tyrosine, D,L-phenylglycine, L-homophenylalanine, and L-DOPA (Figure 7). However, L-kynurenine, an L-tryptophan metabolic degradation product, could be decarboxylated by CaTDC3 (Figure 10). It seems that the consistency of the indole ring of L-tryptophan has little effect on CaTDC3-catalyzed decarboxylation reaction. Closest checking suggested that the structure and conformation of L-kynurenine are similar to those of L-tryptophan (Figures 6A, 12I).

Both chemical and biological approaches had been developed to prepare tryptamine and its analogs (Righi et al., 2012; McDonald et al., 2019; Wang et al., 2019). The present CaTDC3 shows high efficiency in catalytic decarboxylation and displays substrate promiscuity for a wide range of L-tryptophan analogs. Thus, CaTDC3 is an alternative TDC for metabolic engineering and synthetic biological applications (Runguphan et al., 2010; Kalb et al., 2016).

## Conclusion

A TDC-encoding gene, *CaTDC3*, was mined and cloned from camptothecin-producing plant *C. acuminata*. Recombinant CaTDC3 catalyzes an efficiently decarboxylative conversion of L-tryptophan into tryptamine and it shows strict stereoselectivity for L-tryptophan. L-Tryptophan analogs with substituent groups such as hydroxyl and halogen on the indole ring could be recognized by CaTDC3 and its decarboxylation reactions generated the corresponding tryptamines. The C^β^ -methyl-L-tryptophans were decarboxylated by CaTDC3 efficiently. 1-Thio-L-tryptophan, the NH group of the indole ring replaced by an S atom, could be decarboxylated by CaTDC3. CaTDC3 catalyzed the decarboxylation of 7-aza-L-tryptophan, an N displacement of the C atom on the aromatic ring, to give 7-aza-tryptamine. Additionally, L-kynurenine, an L-tryptophan degradation product, could be decarboxylated by CaTDC3. The present works uncover a catalytically promiscuous TDC and it is a versatile decarboxylase in metabolic engineering and synthetic biology for specialized pharmaceutically important substances.

## Data availability statement

The datasets presented in this study can be found in online repositories. The names of the repository/repositories and accession number(s) can be found below: GenBank, ON964510.

## Author contributions

YL conceived and designed the study. CQ, FC, ZL, TH, and WL performed the experimental works. YL and CQ analyzed the data. YL, CQ, and GZ wrote the manuscript. All authors contributed to the article and approved the submitted version.

## Footnotes

1
https://www.ebi.ac.uk/Tools/msa/clustalo/

2
http://www.expasy.ch/tools/protparam.html/

3
http://www.ebi.ac.uk

4
https://www.expasy.org/

5
https://ccsb.scripps.edu/mgltools/
