# Supplementary material for: Functional characterization of a catalytically promiscuous tryptophan decarboxylase from camptothecin-producing Camptotheca acuminata
Source: Front Plant Sci. 2022 Aug 18;13:987348. doi: 10.3389/fpls.2022.987348 (PMC9433702; doi:10.3389/fpls.2022.987348)
Supplement: Supplementary file 1 [file Data_Sheet_1.docx]

***Supplementary Materials* for**

**Functional characterization of a catalytically promiscuous tryptophan decarboxylase from camptothecin-producing *Camptotheca acuminata***

Chong Qiao ^1,2^, Fei Chen ^1^, Zhan Liu ^1,2^, Tianfang Huang ^1^, Wei Li ^1^, Guolin Zhang ^1^, Yinggang Luo ^1,^*

^1^ Center for Natural Products Research, Chengdu Institute of Biology, Chinese Academy of Sciences, 9 Section 4, Renmin Road South, Chengdu 610041, People’s Republic of China

^2^ University of Chinese Academy of Sciences, 19A Yuquan Road, Beijing 100049, People’s Republic of China

*To whom correspondence should be addressed: Yinggang Luo, Center for Natural Products Research, Chengdu Institute of Biology, Chinese Academy of Sciences, Chengdu 610041, China; E-mail: yinggluo@cib.ac.cn; Tel.: +86 28 8289 0813; Fax: +86 28 8289 0288

**Contents**

**Supplementary Figure S1………………………………………………………………………….……...3**

**Supplementary Figure S2………………………………………………………………………….……...4**

**Supplementary Figure S3………………………………………………………………………….……...5**

**Supplementary Figure S4………………………………………………………………………….……...7**

**Supplementary Figure S5………………………………………………………………………….……...8**

**Supplementary Figure S6………………………………………………………………………….……...9**

**Supplementary Figure S7……………………………………………………………………….……...10**

**Supplementary Figure S8……………………………………………………………………….……...11**

**Supplementary Figure S9……………………………………………………………………….……...12**

**Supplementary Figure S10……………………………………………………………………….…….13**

**Supplementary Figure S11……………………………………………………………………….…….14**

**Supplementary Figure S12……………………………………………………………………….…….15**

**Supplementary Figure S13……………………………………………………………………….…….16**

**Supplementary Figure S14……………………………………………………………………….…….17**

**Supplementary Figure S15……………………………………………………………………….…….18**

**Supplementary Figure S16……………………………………………………………………….…….19**

**Supplementary Figure S17……………………………………………………………………….…….20**

**Supplementary Figure S18……………………………………………………………………….…….21**

**Supplementary Figure S19……………………………………………………………………….…….22**

**Supplementary Figure S20……………………………………………………………………….…….23**

**Supplementary Figure S21……………………………………………………………………….…….24**

**Supplementary Figure S22……………………………………………………………………….…….25**

**Supplementary Figure S23……………………………………………………………………….…….26**

**Supplementary Figure S24……………………………………………………………………….…….27**


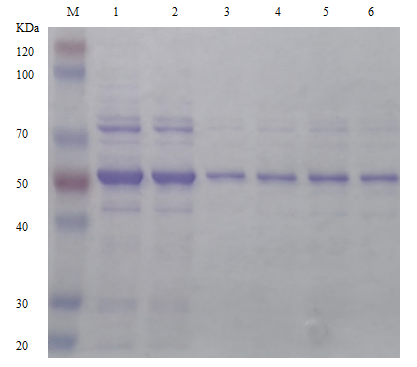


**Supplementary Figure S1.** SDS-PAGE analyses of overexpression and purification of CaTDC3. M, protein marker; Lanes 1 - 6, the eluent fractions eluted with elution buffer containing 250 mM imidaozole from the Nickel-nitrolotriacetic acid resin. The target protein band was indicated with a red arrow.


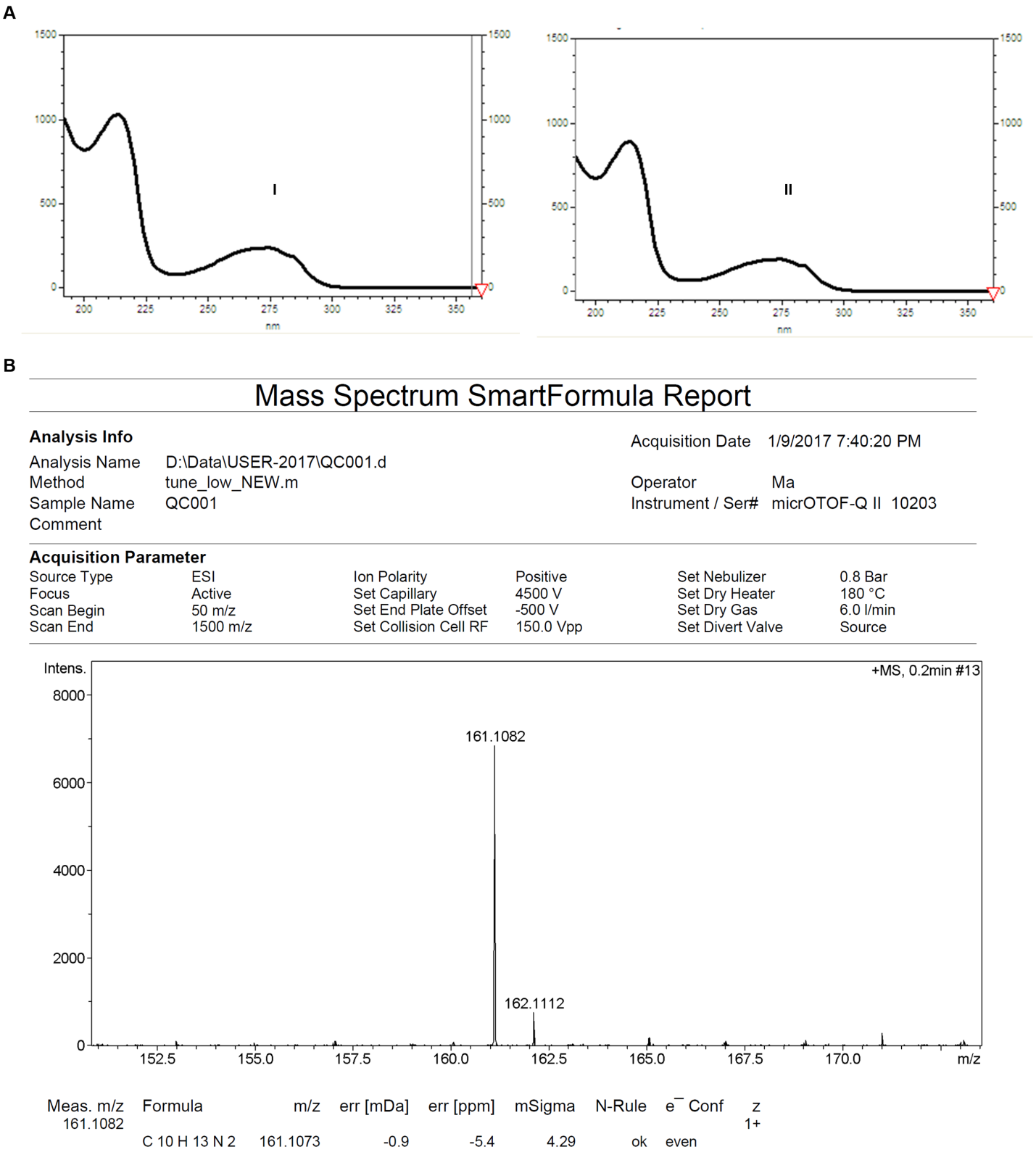


**Supplementary Figure S2.**  **A**, The UV spectra of the enzymatic conversion product from CaTDC3 (*panel* **Ⅰ**) and the standard tryptamine (*panel* **Ⅱ**). **B**, HRMS(ESI) of the enzymatic conversion product.


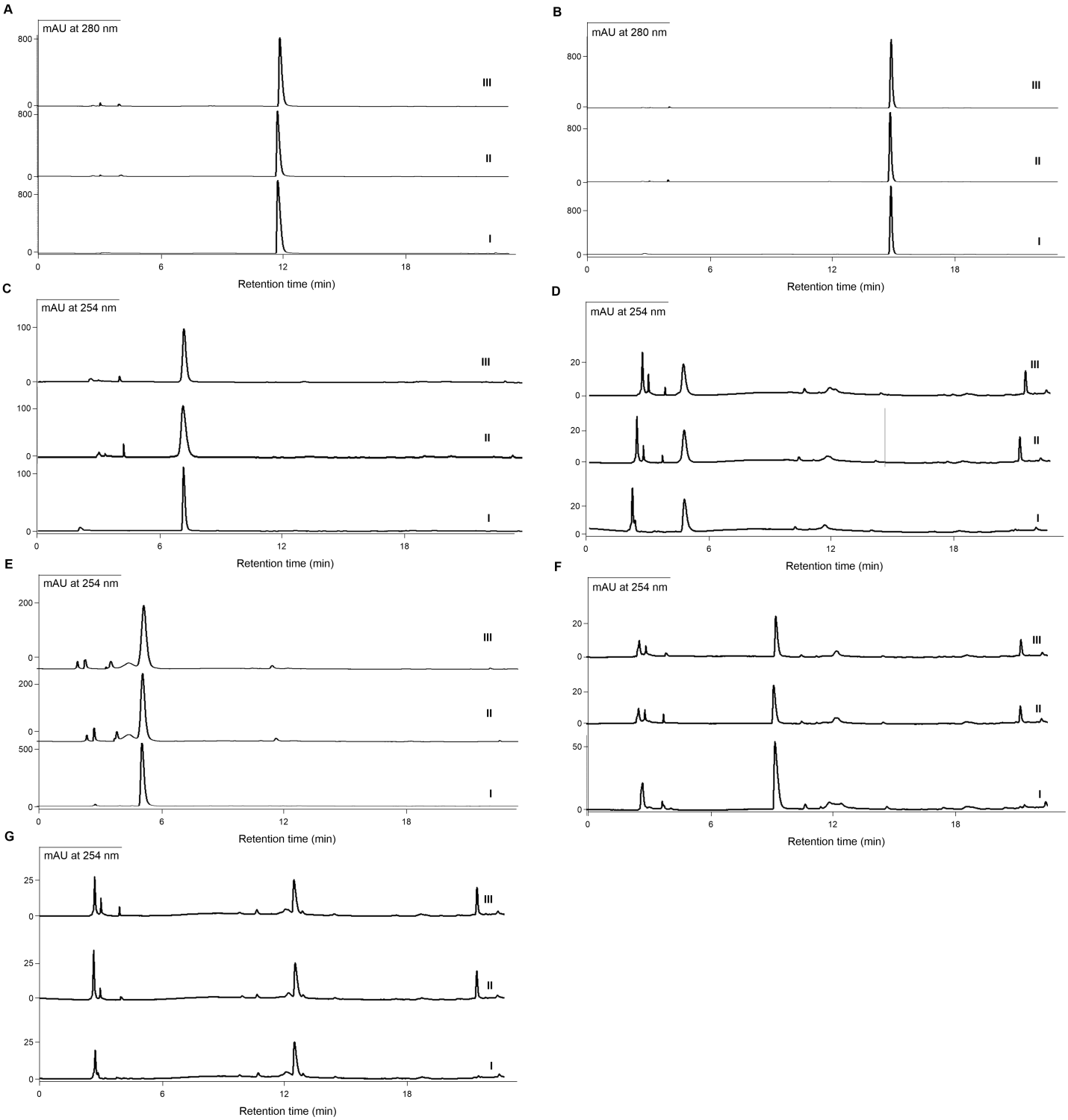


**Supplementary Figure S3.**  CaTDC3-catalyzed reactions towards L-tryptophan analogues with different substituted aromatic rings and side chains. **A**, HPLC-DAD analyses of the standard D-tryptophan (*panel* **Ⅰ**), the reaction mixture with CaTDC3 (*panel* **II**) and boiled CaTDC3 (*panel* **III**) as catalyst, respectively, monitored at 280 nm. **B**, HPLC-DAD analyses of the standard N^α^-acetyl-L-tryptophan (*panel* **Ⅰ**), the reaction mixture with CaTDC3 (*panel* **II**) and boiled CaTDC3 (*panel* **III**) as catalyst, respectively, monitored at 280 nm. **C**, HPLC-DAD analyses of the standard L-tyrosine (*panel* **Ⅰ**), the reaction mixture with CaTDC3 (*panel* **II**) and boiled CaTDC3 (*panel* **III**) as catalyst, respectively, monitored at 254 nm. **D**, HPLC-DAD analyses of the standard D,L-phenylglycine (*panel* **Ⅰ**), the reaction mixture with CaTDC3 (*panel* **II**) and boiled CaTDC3 (*panel* **III**) as catalyst, respectively, monitored at 254 nm. **E**, HPLC-DAD analysis of the standard L-dihydroxyphenylalanine (*panel* **Ⅰ**), the reaction mixture with CaTDC3 (*panel* **II**) and boiled CaTDC3 (*panel* **III**) as catalyst, respectively, monitored at 254 nm. **F**, HPLC-DAD analyses of the standard L-phenylalanine (*panel* **Ⅰ**), the reaction mixture with CaTDC3 (*panel* **II**) and boiled CaTDC3 (*panel* **III**) as catalyst, respectively, monitored at 254 nm. **G**, HPLC-DAD analyses of the standard L-homophenylalanine (*panel* **Ⅰ**), the reaction mixture with CaTDC3 (*pane*l **II**) and boiled CaTDC3 (*pane*l **III**) as catalyst, respectively, monitored at 254 nm.


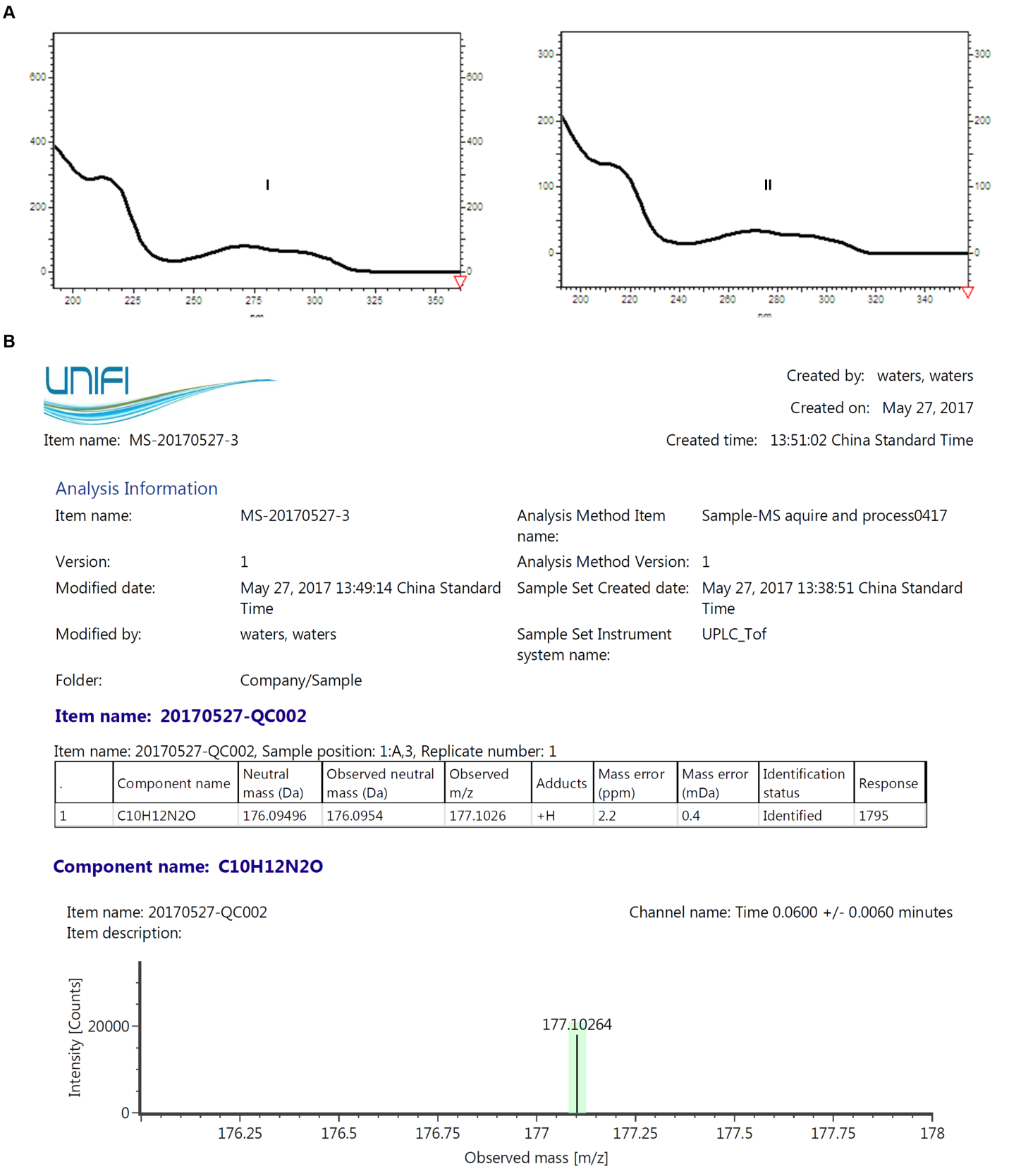


**Supplementary Figure S4.** **A**, The UV spectra of the enzymatic conversion product from CaTDC3 (*panel* **Ⅰ**) and the standard 5-hydroxytryptamine (*panel* **Ⅱ**). **B**, HRMS(ESI) of the enzymatic conversion product.


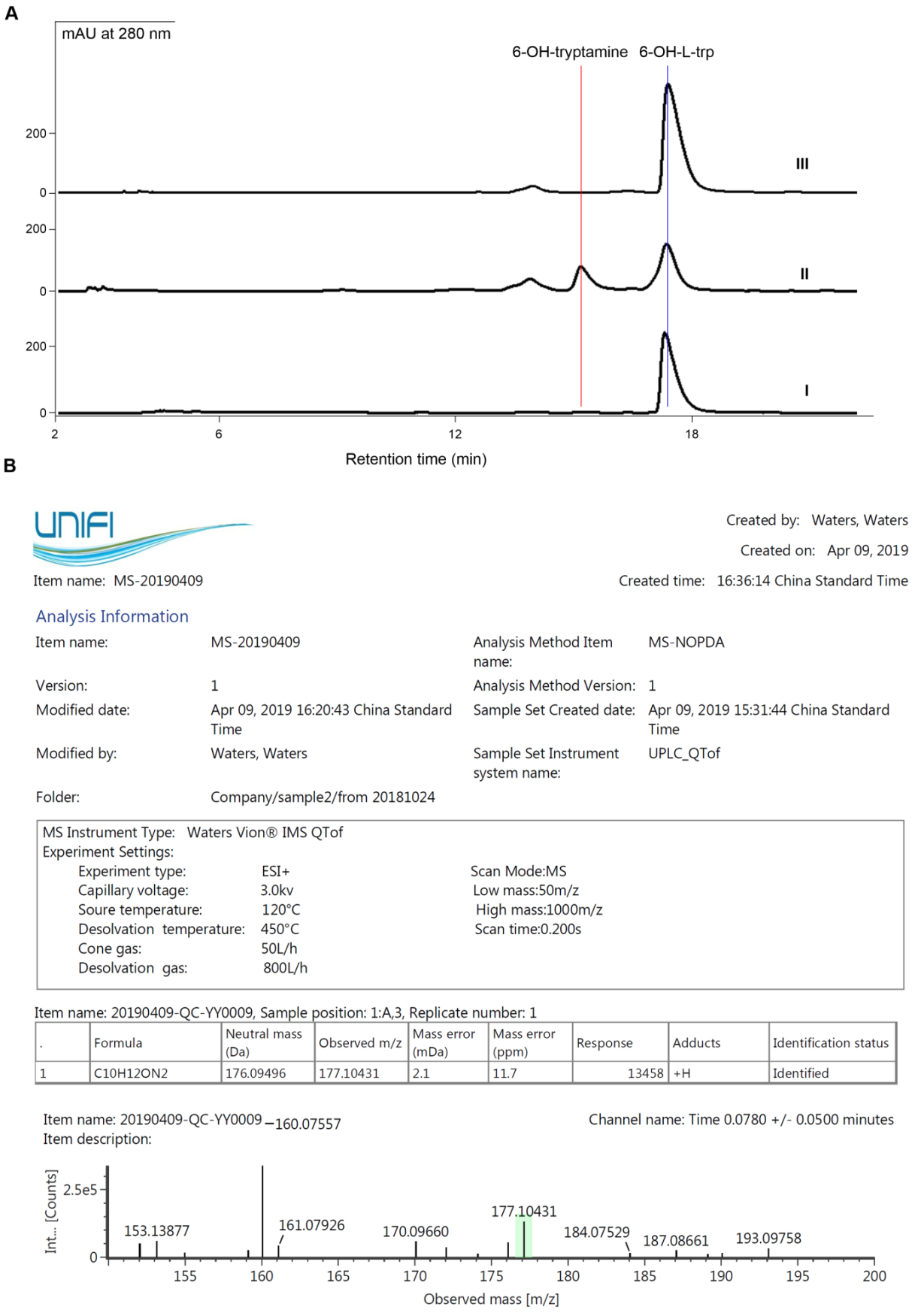


**Supplementary Figure S5.**  CaTDC3-catalyzed decarboxylation using 6-hydroxy-L-tryptophan as substrate. **A**, HPLC-DAD analyses the standard 6-hydroxy-L-tryptophan (*panel* **Ⅰ**), the enzymatic reaction mixture with CaTDC3 (*panel* **Ⅱ**) and boiled CaTDC3 (*panel* **Ⅲ**) as catalyst, respectively, monitored at 280 nm. **B**, HRMS(ESI) of the enzymatic conversion product.


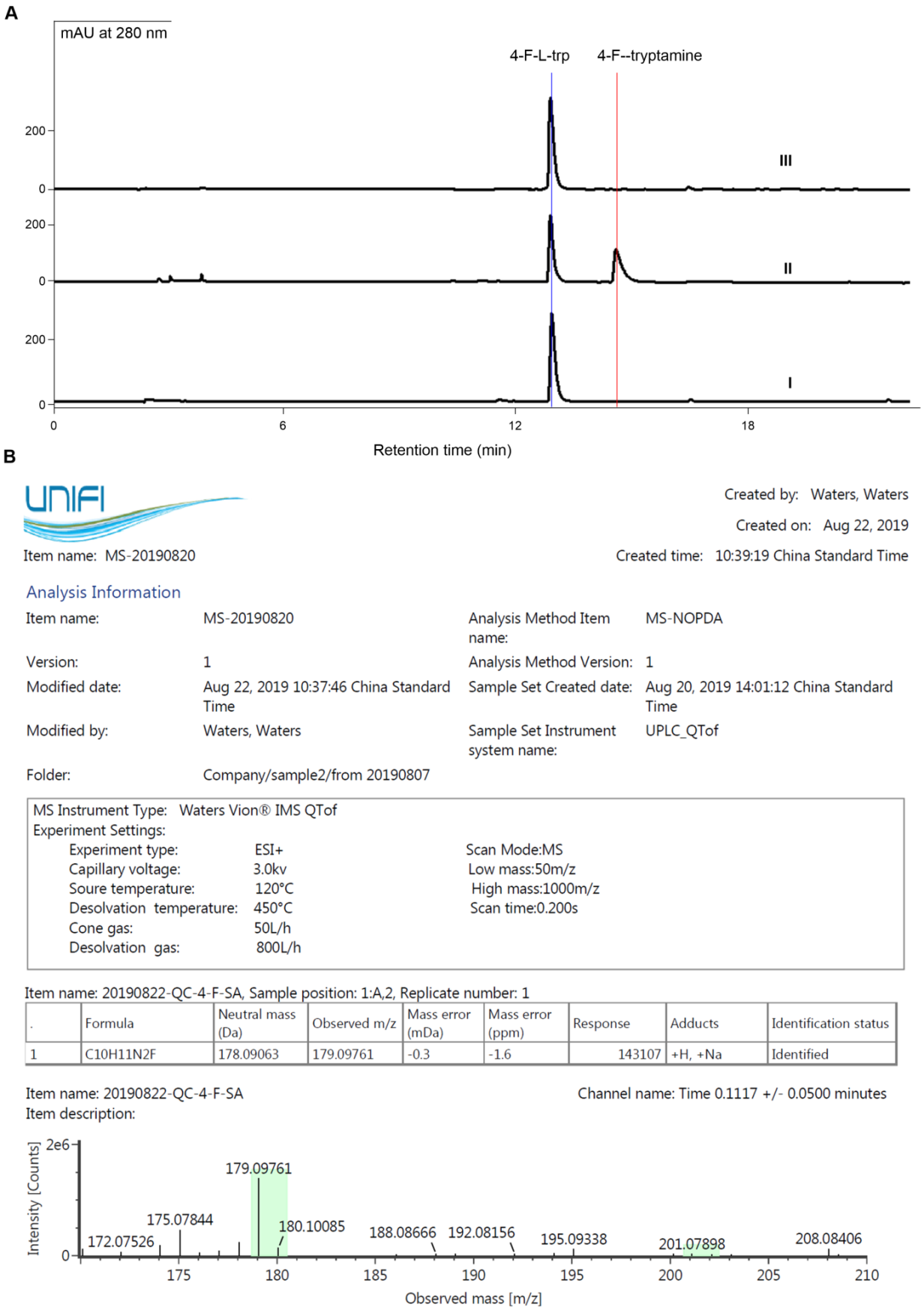


**Supplementary Figure S6.** CaTDC3-catalyzed decarboxylation using 4-fluoro-L-tryptophan as substrate. **A**, HPLC-DAD analyses the standard 4-fluoro-L-tryptophan (*panel* **Ⅰ**), the enzymatic reaction mixture with CaTDC3 (*panel* **Ⅱ**) and boiled CaTDC3 (*panel* **Ⅲ**) as catalyst, respectively, monitored at 280 nm. **B**, HRMS(ESI) of the enzymatic conversion product.


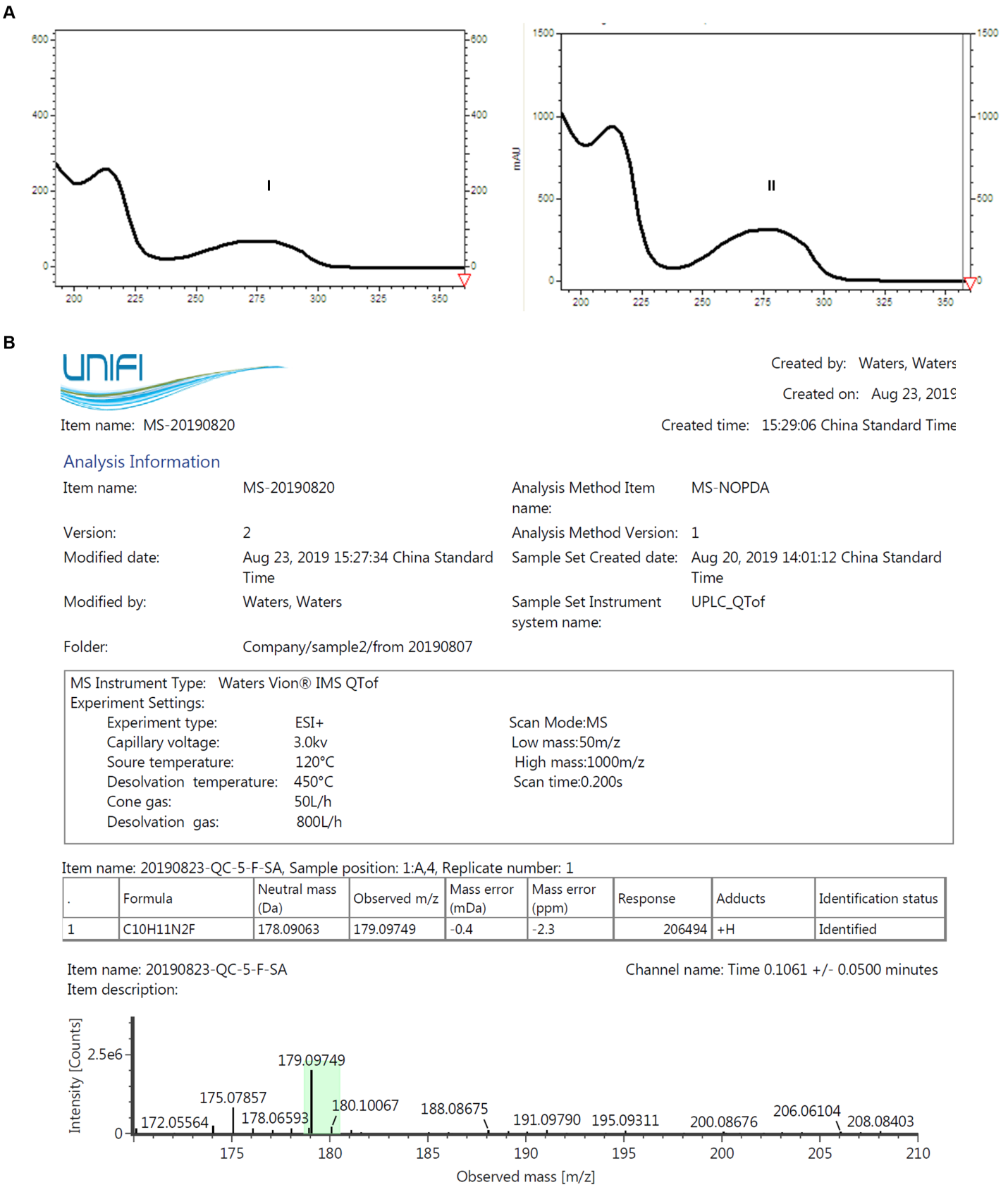


**Supplementary Figure S7.** **A**, The UV spectra of the enzymatic conversion product from CaTDC3 (*panel* **Ⅰ**) and the standard 5-fluorotryptamine (*panel* **Ⅱ**). **B**, HRMS(ESI) of the enzymatic conversion product.


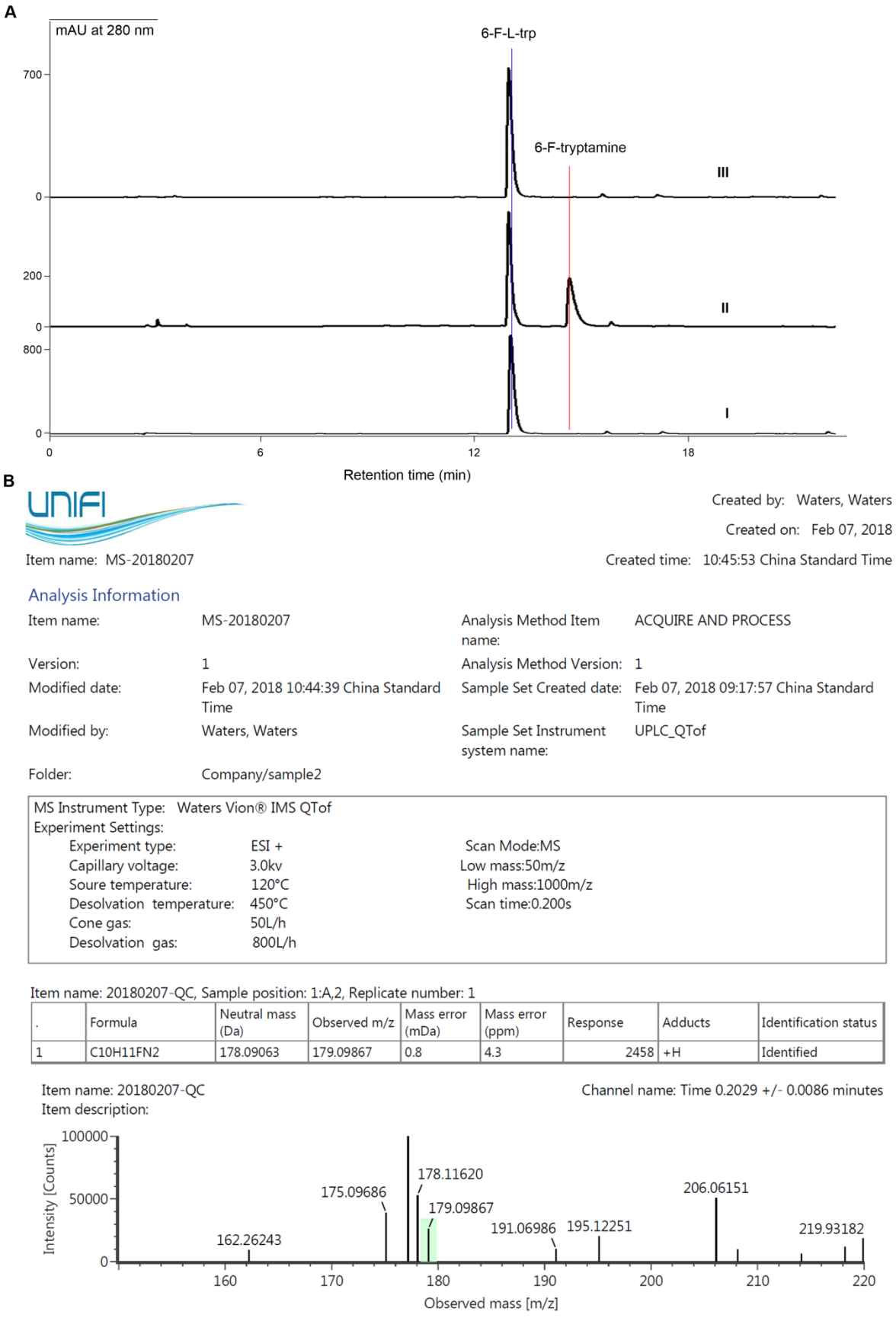


**Supplementary Figure S8.** CaTDC3-catalyzed decarboxylation using 6-fluoro-L-tryptophan as substrate. **A**, HPLC-DAD analyses the standard 6-fluoro-L-tryptophan (*panel* **Ⅰ**), the enzymatic reaction mixture with CaTDC3 (*panel* **Ⅱ**) and boiled CaTDC3 (*panel* **Ⅲ**) as catalyst, respectively, monitored at 280 nm. **B**, HRMS(ESI) of the enzymatic conversion product.


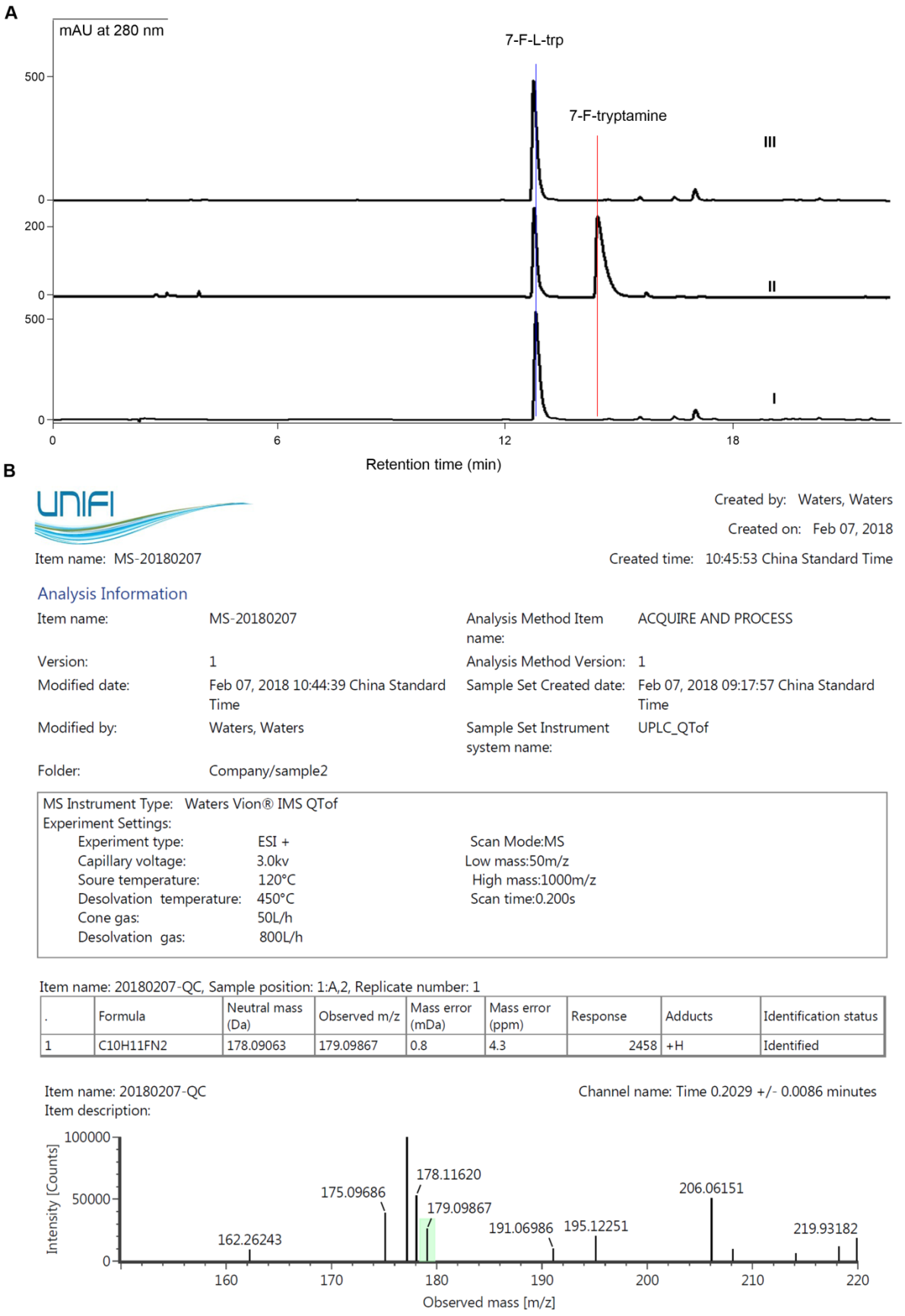


**Supplementary Figure S9.** CaTDC3-catalyzed decarboxylation using 7-fluoro-L-tryptophan as substrate. **A**, HPLC-DAD analyses the standard 7-fluoro-L-tryptophan (*panel* **Ⅰ**), the enzymatic reaction mixture with CaTDC3 (*panel* **Ⅱ**) and boiled CaTDC3 (*panel* **Ⅲ**) as catalyst, respectively, monitored at 280 nm. **B**, HRMS(ESI) of the enzymatic conversion product.


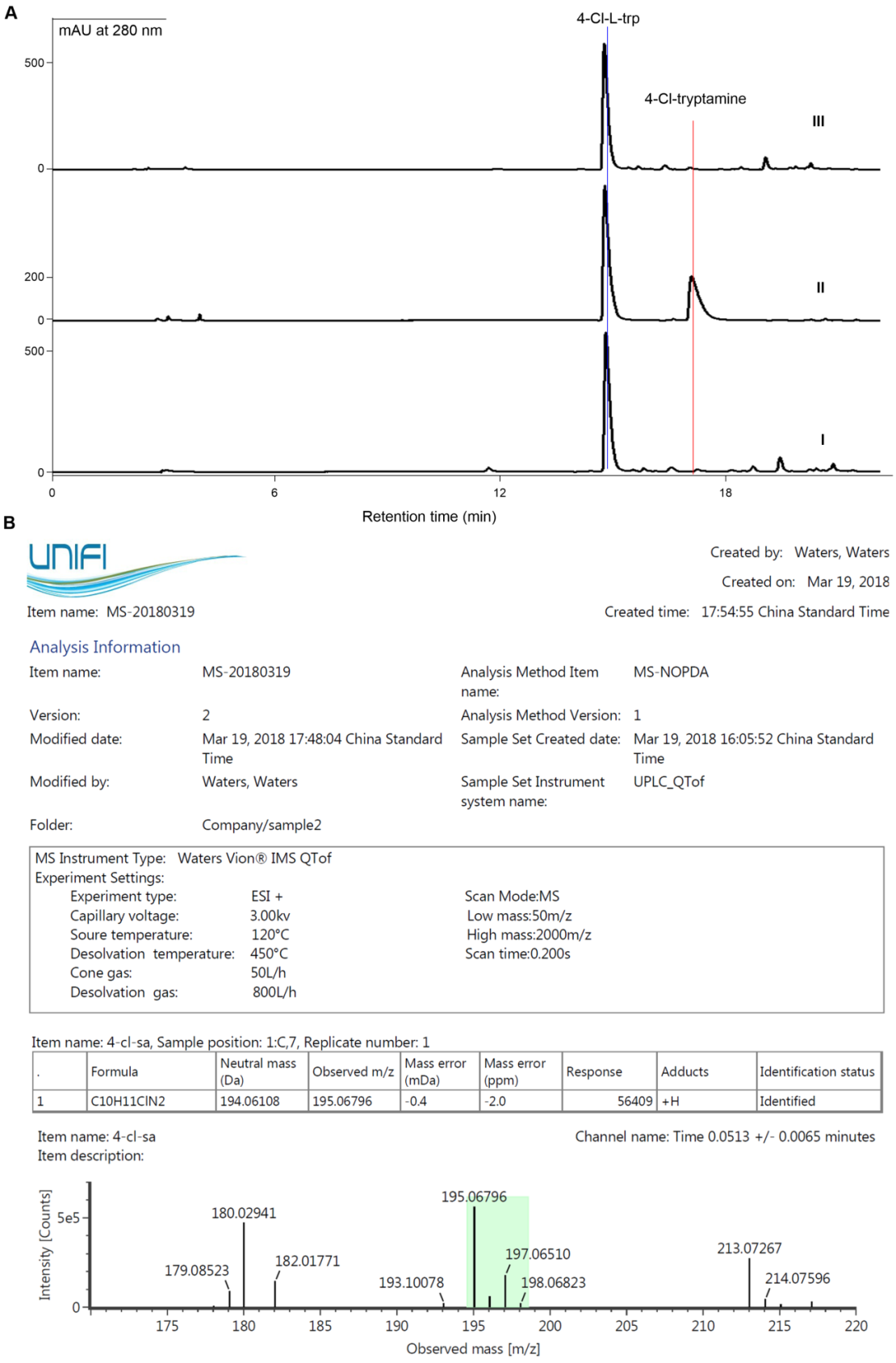


**Supplementary Figure S10.** CaTDC3-catalyzed decarboxylation using 4-chloro-L-tryptophan as substrate. **A**, HPLC-DAD analyses the standard 4-chloro-L-tryptophan (*panel* **Ⅰ**), the enzymatic reaction mixture with CaTDC3 (*panel* **Ⅱ**) and boiled CaTDC3 (*panel* **Ⅲ**) as catalyst, respectively, monitored at 280 nm. **B**, HRMS(ESI) of the enzymatic conversion product.


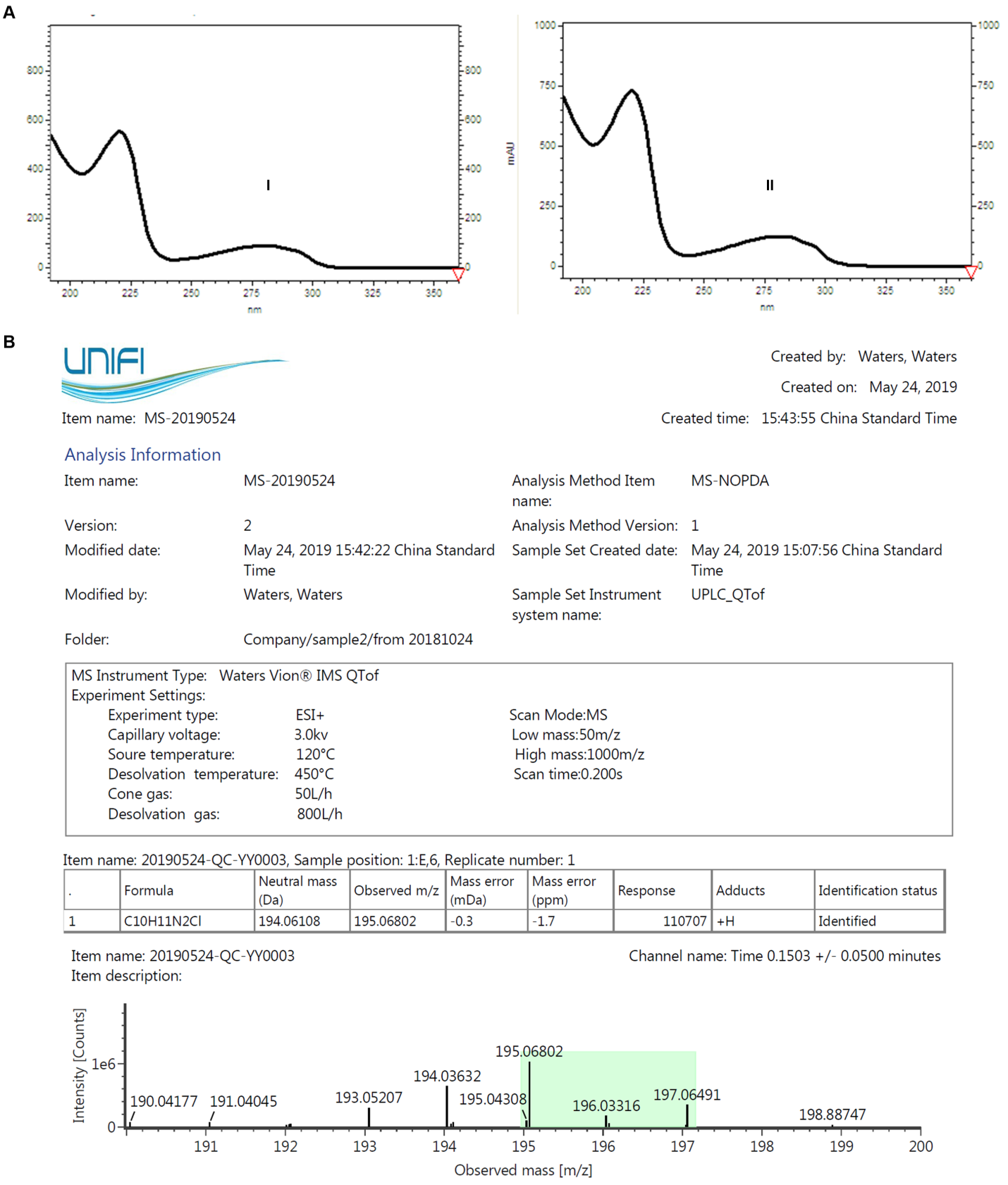


**Supplementary Figure S11.** **A**, The UV spectra of the enzymatic conversion product from CaTDC3 (*panel* **Ⅰ**) and the standard 5-chlorotryptamine (*panel* **Ⅱ**). **B**, HRMS(ESI) of the enzymatic conversion product.


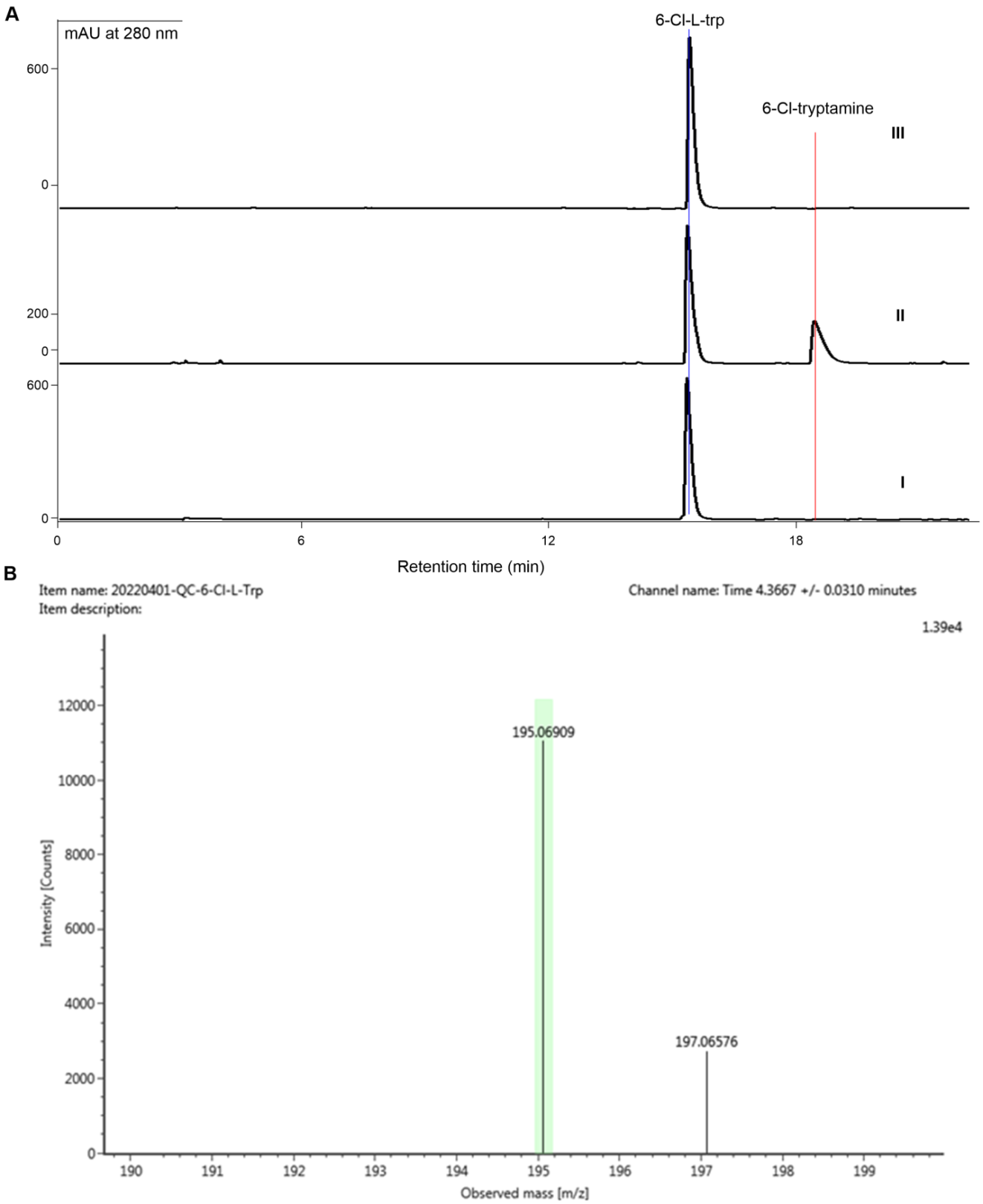


**Supplementary Figure S12.** CaTDC3-catalyzed decarboxylation using 6-chloro-L-tryptophan as substrate. **A**, HPLC-DAD analyses the standard 6-chloro-L-tryptophan (*panel* **Ⅰ**), the enzymatic reaction mixture with CaTDC3 (*panel* **Ⅱ**) and boiled CaTDC3 (*panel* **Ⅲ**) as catalyst, respectively, monitored at 280 nm. **B**, HRMS(ESI) of the enzymatic conversion product.


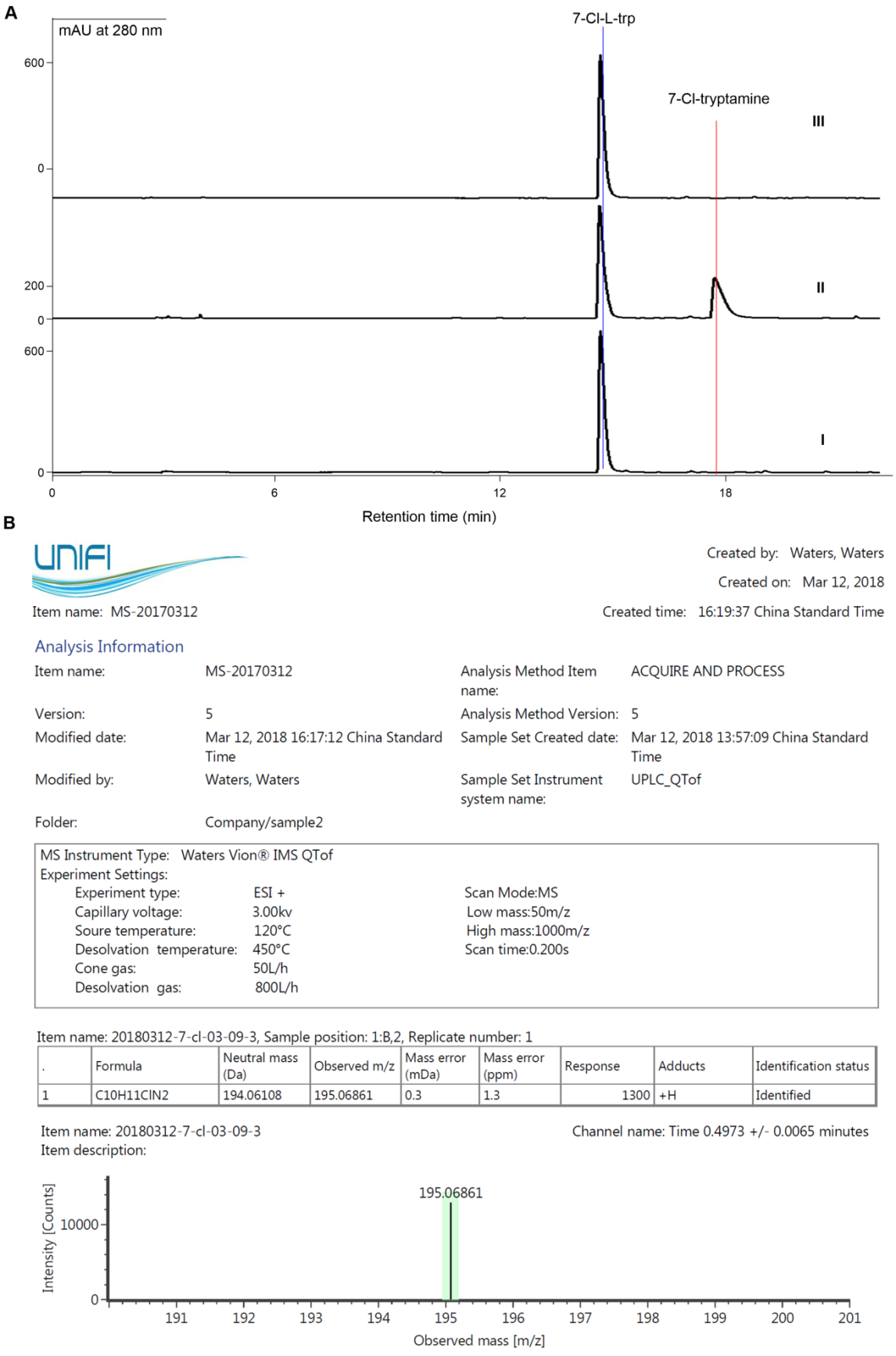


**Supplementary Figure S13.** CaTDC3-catalyzed decarboxylation using 7-chloro-L-tryptophan as substrate. **A**, HPLC-DAD analyses the standard 7-chloro-L-tryptophan (*panel* **Ⅰ**), the enzymatic reaction mixture with CaTDC3 (*panel* **Ⅱ**) and boiled CaTDC3 (*panel* **Ⅲ**) as catalyst, respectively, monitored at 280 nm. **B**, HRMS(ESI) of the enzymatic conversion product.


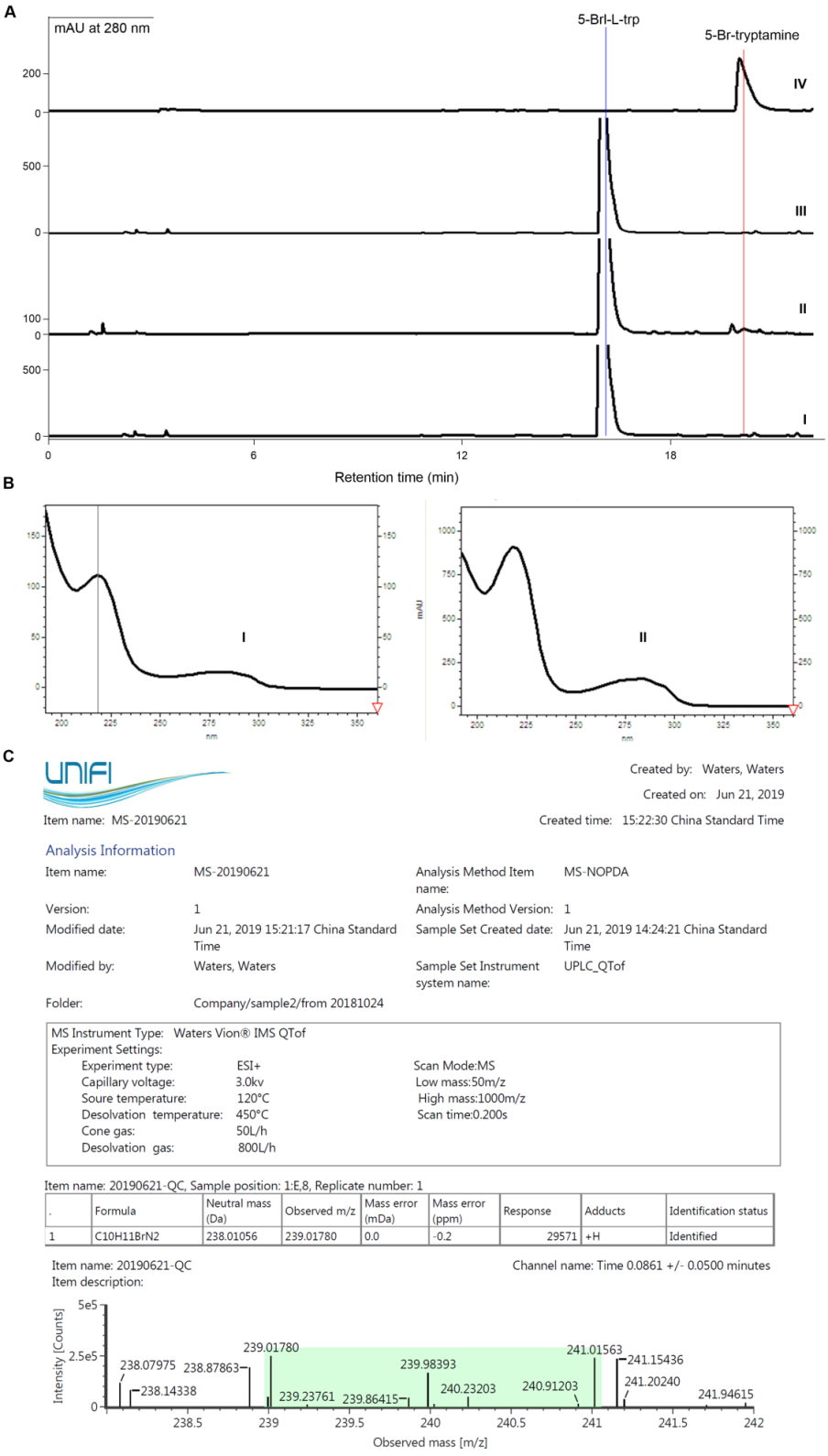


**Supplementary Figure S14.** CaTDC3-catalyzed decarboxylation using 5-bromo-L-tryptophan as substrate. **A**, HPLC-DAD analyses the standard 5-bromo-L-tryptophan (*panel* **Ⅰ**), the enzymatic reaction mixture with CaTDC3 (*panel* **Ⅱ**) and boiled CaTDC3 (*panel* **Ⅲ**) as catalyst, respectively, and the standard 5-bromotryptamine, monitored at 280 nm. **B**, The UV spectra of the enzymatic conversion product from CaTDC3 (*panel* **Ⅰ**) and the standard 5-bromotryptamine (*panel* **Ⅱ**). **C**, HRMS(ESI) of the enzymatic conversion product.


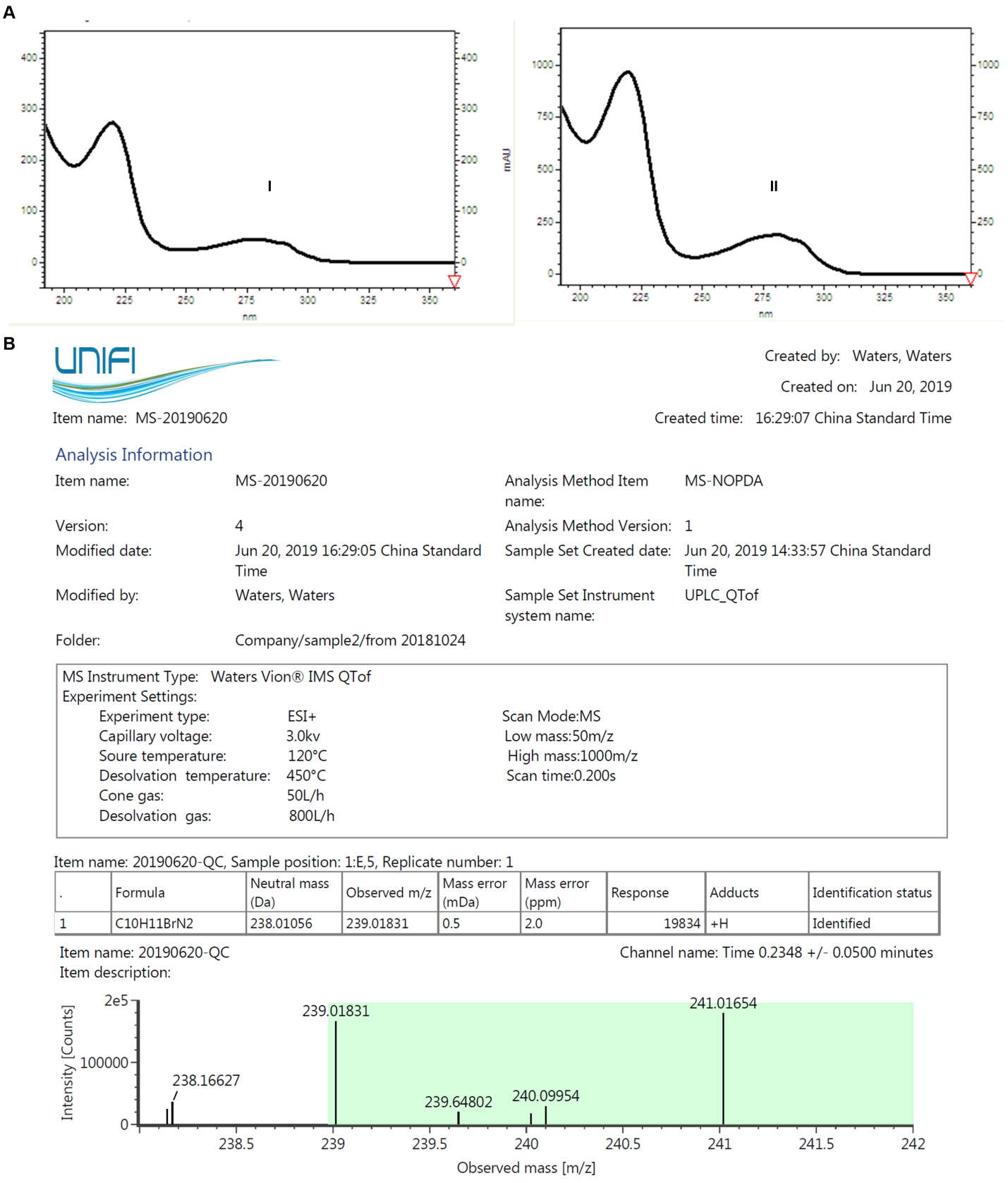


**Supplementary Figure S15. A**, The UV spectra of the enzymatic conversion product from CaTDC3 (*panel* **Ⅰ**) and the standard 6-bromotryptamine (*panel* **Ⅱ**). **B**, HRMS(ESI) of the enzymatic conversion product.


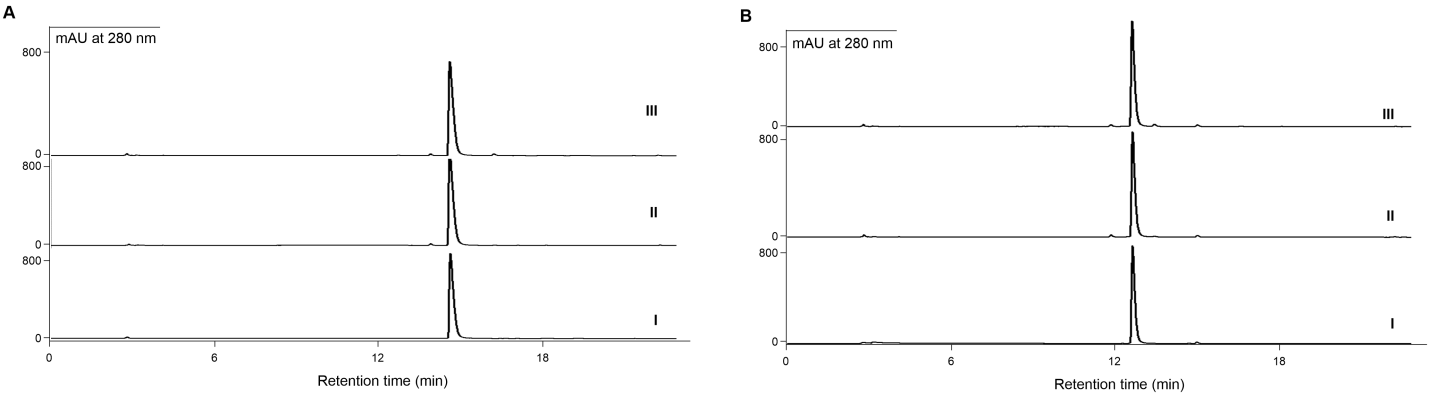


**Supplementary Figure S16.** CaTDC3-catalyzed reactions towards 5-methyl- and 5-methoxy-L-tryptophans (**A** and **B**, respectively). HPLC-DAD analyses of the standards (*panel*s **Ⅰ**, 5-methyl-L-tryptophan, **A**; 5-methoxy-L-tryptophan, **B**), the reaction mixture with CaTDC3 (*panel*s **II**) and boiled CaTDC3 (*panel*s **III**) as catalyst, respectively, monitored at 280 nm.


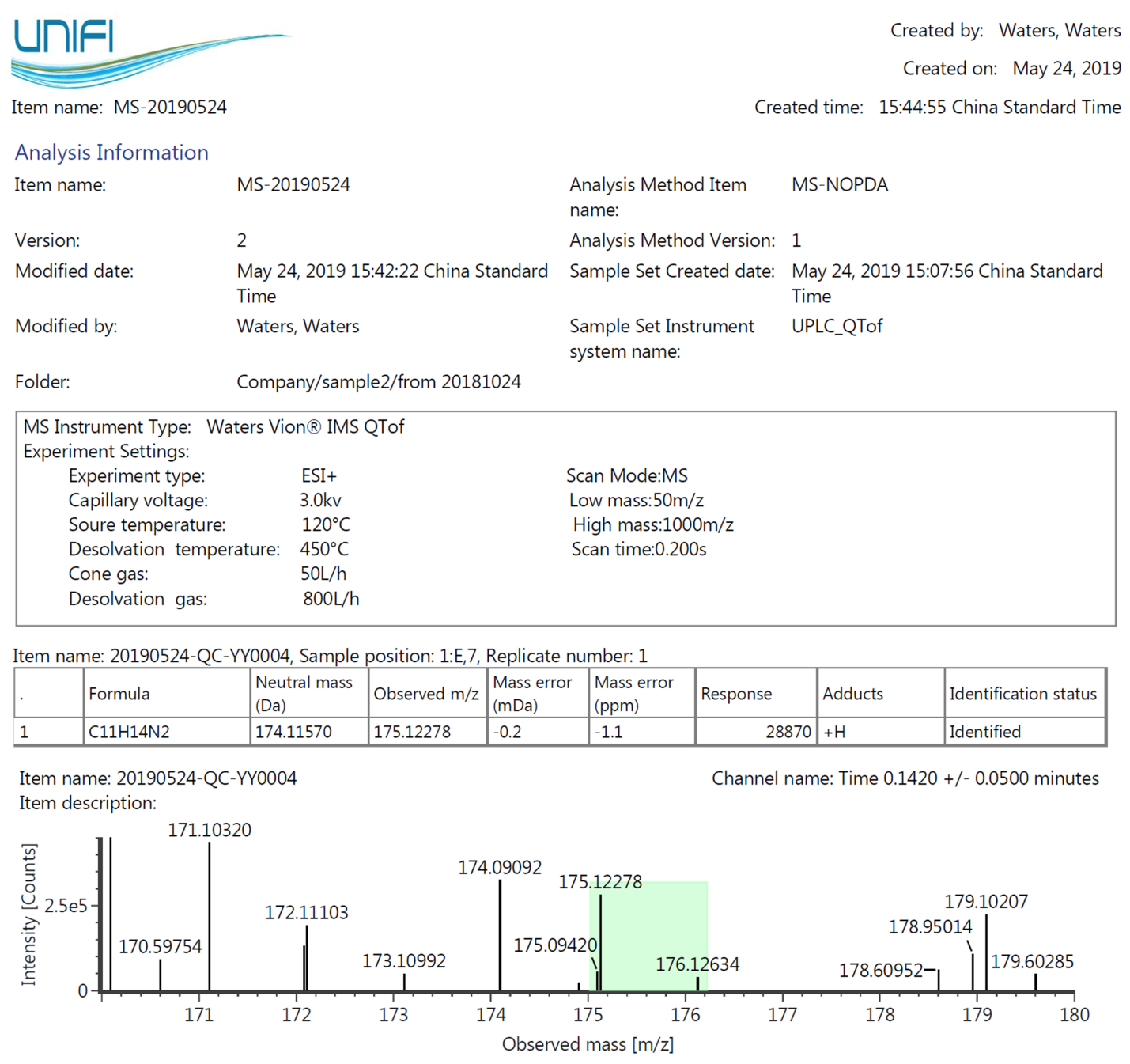


**Supplementary Figure S17.** HRMS(ESI) of the enzymatic conversion product using (*R*)-C^β^-methyl- and (*S*)-C^β^-methyl-L-tryptophans as substrate.


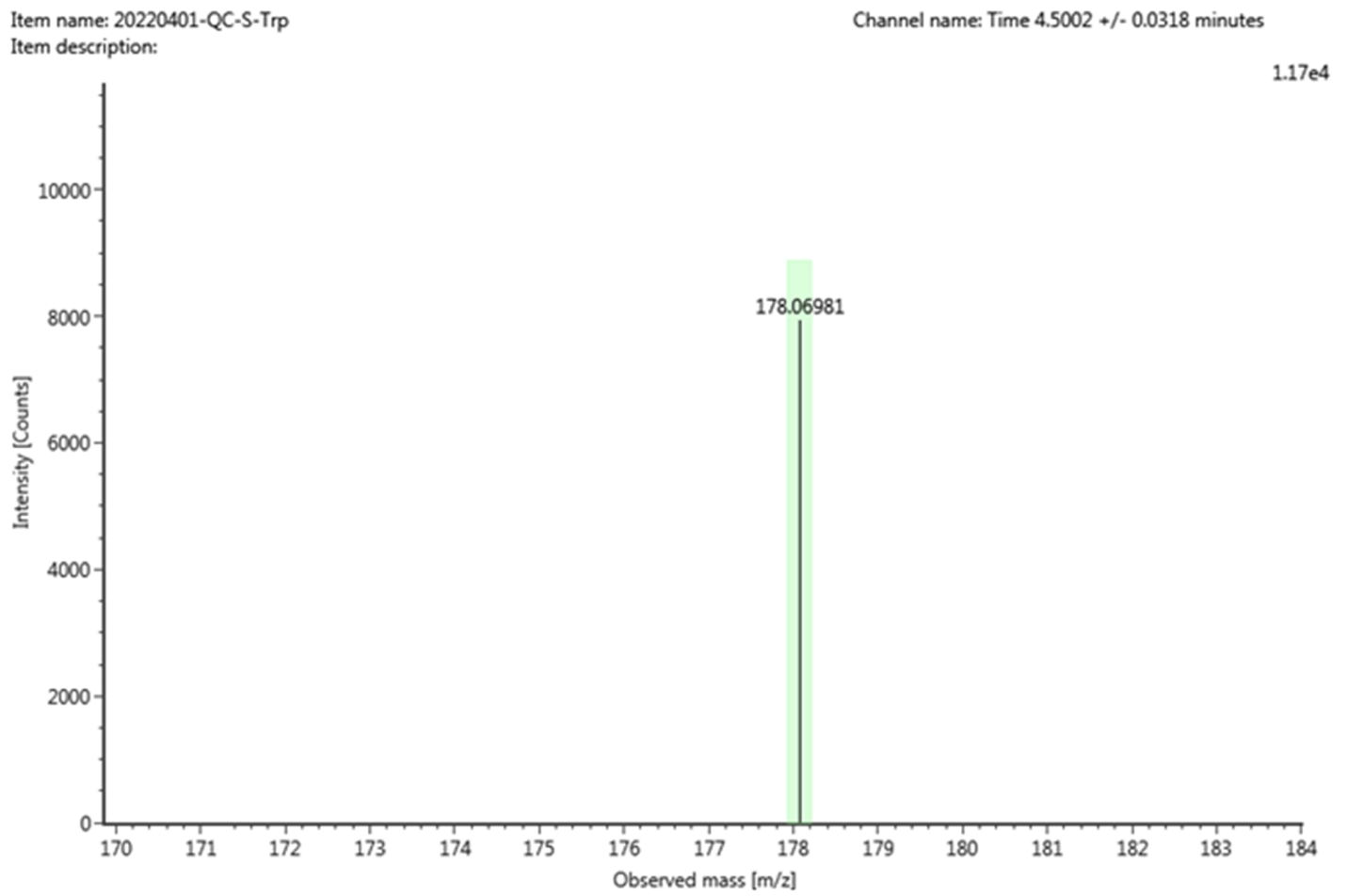


**Supplementary Figure S18.** HRMS(ESI) of the enzymatic conversion product using 1-thio-L-tryptophan as substrate.

**Supplementary Figure S19.** ^1^H-NMR spectrum of 1-thio-tryptamine (δ_H_ 0.0 ~ 8.5)

**Supplementary Figure S20.** ^1^H-NMR spectrum of 1-thio-tryptamine (δ_H_ 7.20 ~ 8.15)

**Supplementary Figure S21.** ^1^H-NMR spectrum of 1-thio-tryptamine (δ_H_ 3.10 ~ 3.31)


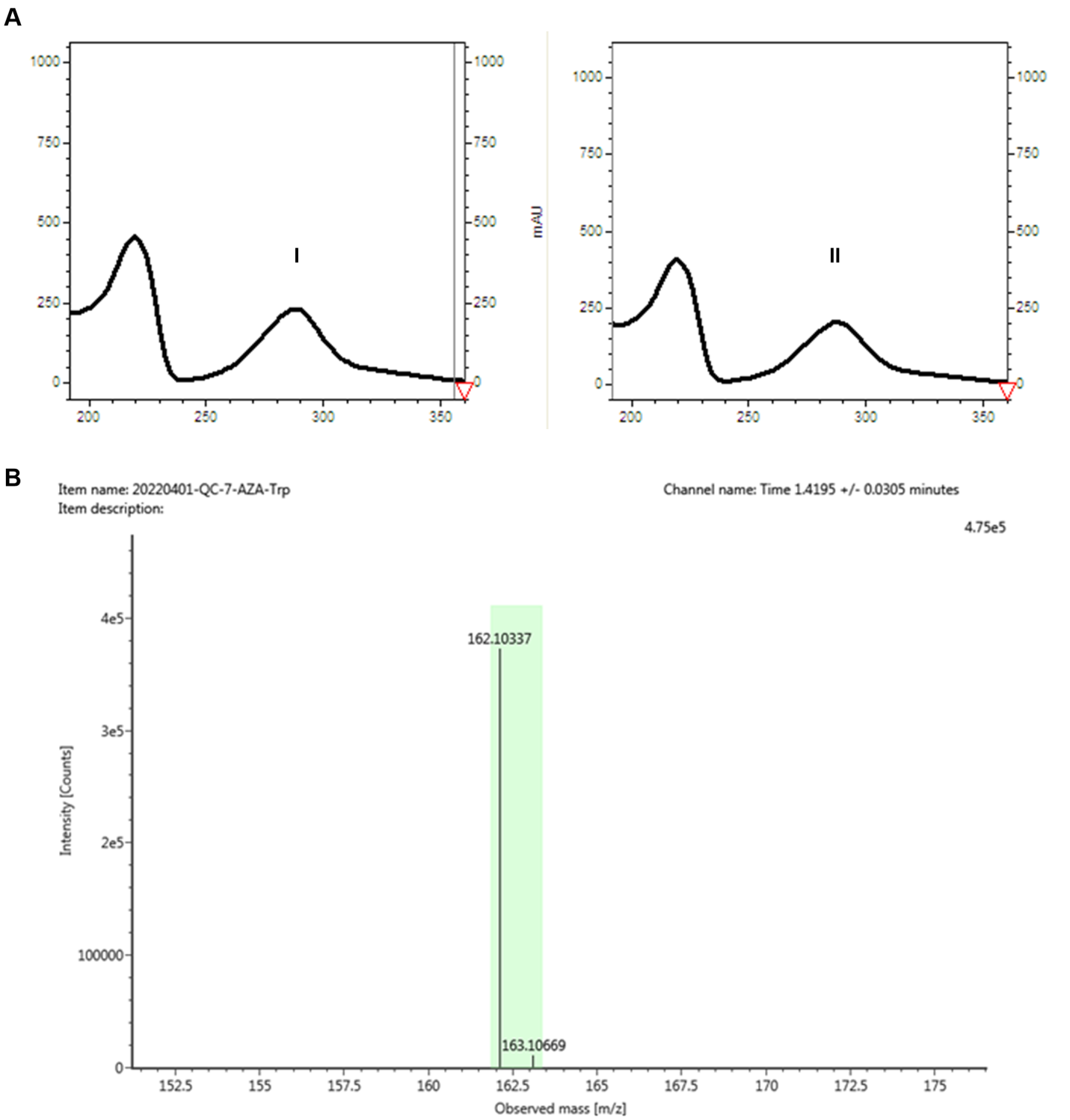


**Supplementary Figure S22.** **A**, The UV spectra of the enzymatic conversion product from CaTDC3 (*panel* **Ⅰ**) and the standard 7-aza-tryptamine (*panel* **Ⅱ**). **B**, HRMS(ESI) of the enzymatic conversion product.


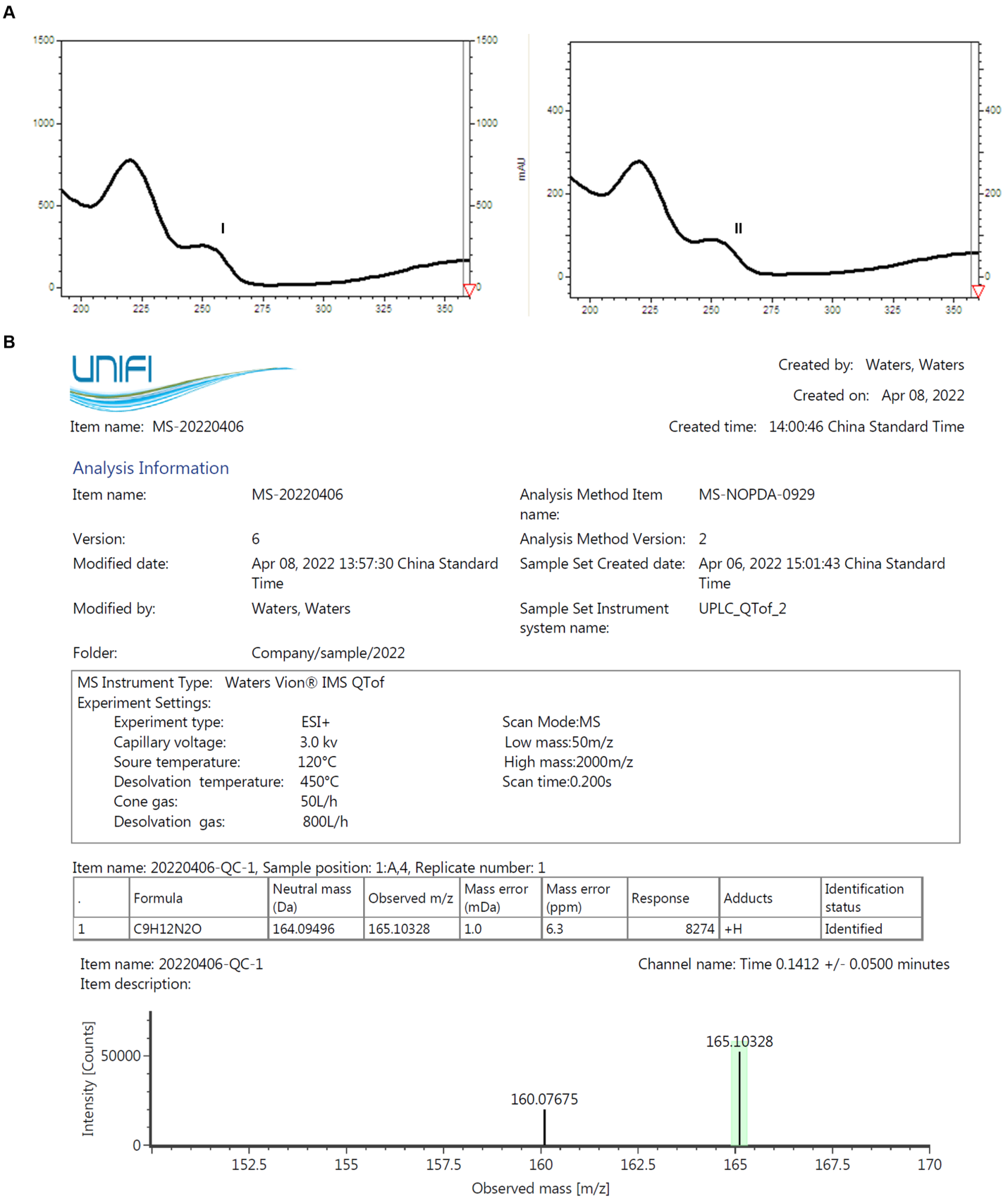


**Supplementary Figure S23. A**, The UV spectra of the enzymatic conversion product from CaTDC3 (*panel* **Ⅰ**) and the standard kynuramine (*panel* **Ⅱ**). **B**, HRMS(ESI) of the enzymatic conversion product.


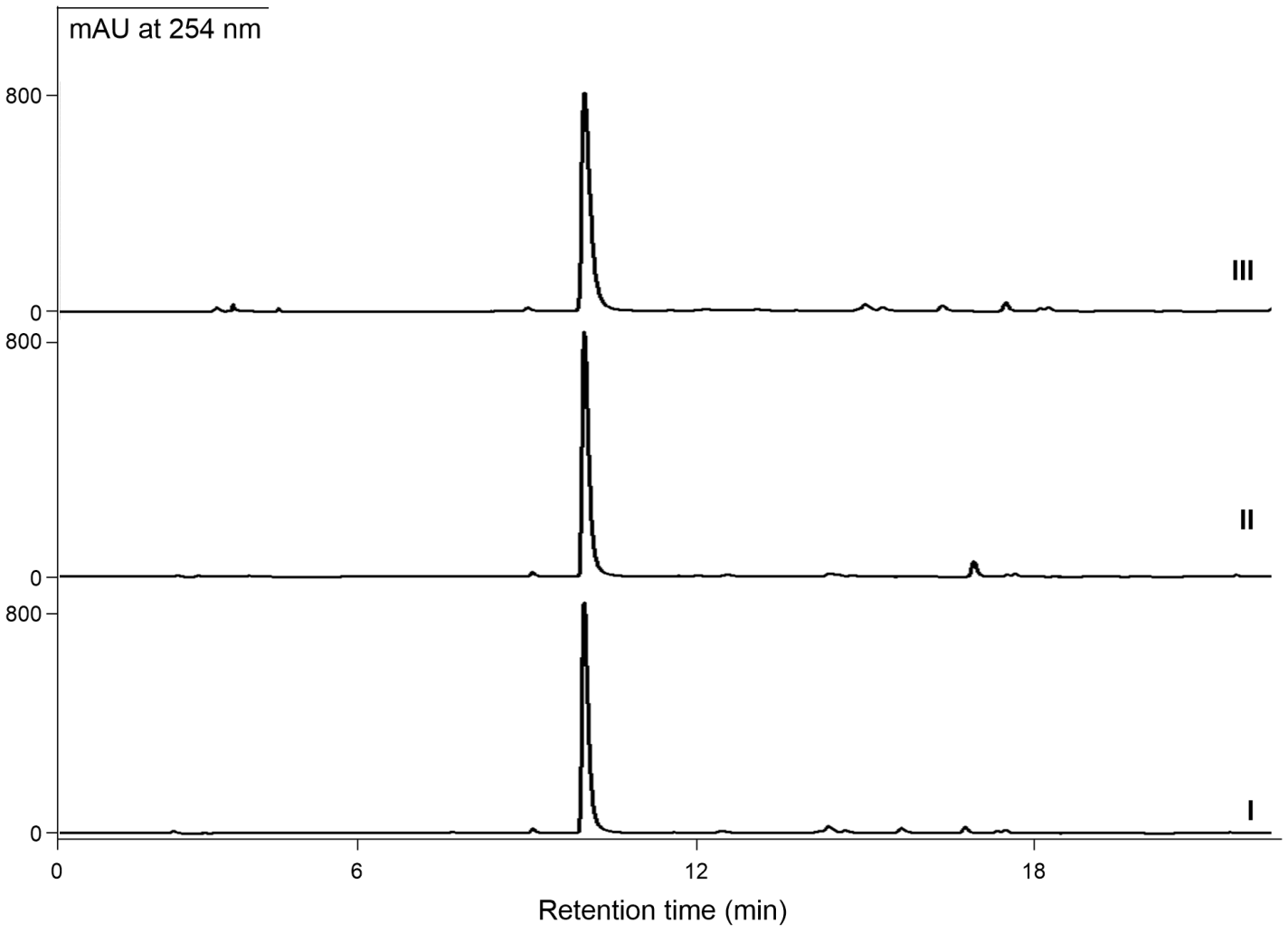


**Supplementary Figure S24.** CaTDC3-catalyzed reaction towards D-kynurenine. HPLC-DAD analyses of the standard D-kynurenine (*panel* **Ⅰ**), the reaction mixture with CaTDC3 (*panel*s **II**) and boiled CaTDC3 (*panel*s **III**) as catalyst, respectively, monitored at 254 nm.
